# A Common Mechanism Underlying Food Choice and Social Decisions

**DOI:** 10.1371/journal.pcbi.1004371

**Published:** 2015-10-13

**Authors:** Ian Krajbich, Todd Hare, Björn Bartling, Yosuke Morishima, Ernst Fehr

**Affiliations:** 1 Department of Economics, University of Zurich, Zurich, Switzerland; 2 Department of Psychology, The Ohio State University, Columbus, Ohio, United States of America; 3 Department of Economics, The Ohio State University, Columbus, Ohio, United States of America; 4 Translational Research Center, University Hospital of Psychiatry, University of Bern, Bern, Switzerland; 5 Japan Science and Technology Agency, PRESTO, Saitama, Japan; Brain and Spine Institute (ICM), FRANCE

## Abstract

People make numerous decisions every day including perceptual decisions such as walking through a crowd, decisions over primary rewards such as what to eat, and social decisions that require balancing own and others’ benefits. The unifying principles behind choices in various domains are, however, still not well understood. Mathematical models that describe choice behavior in specific contexts have provided important insights into the computations that may underlie decision making in the brain. However, a critical and largely unanswered question is whether these models generalize from one choice context to another. Here we show that a model adapted from the perceptual decision-making domain and estimated on choices over food rewards accurately predicts choices and reaction times in four independent sets of subjects making social decisions. The robustness of the model across domains provides behavioral evidence for a common decision-making process in perceptual, primary reward, and social decision making.

## Author Summary

One critical question that concerns all disciplines involved in the study of human decision-making is whether different types of decisions are made in different ways, or whether there exists a common decision mechanism that underlies human choices. If the latter, what are the properties of that mechanism? Here we characterize a dynamical model of decision making that was initially fit to subjects making food choices but was later able to accurately predict choices and reaction times of separate groups of subjects making social decisions. The robustness of the model across different subjects, tasks, and environments supports the idea that the brain uses a consistent process for making decisions.

## Introduction

The most basic tenet in standard economic theory is that individuals know their preferences, and those preferences in turn determine their choices. In this approach, preference and choice are one and the same thing. However, this fundamental assumption is at odds with an extensive experimental literature documenting noisy and inconsistent choices, sometimes referred to as preference reversals [1–4]. Based on these experimental findings, theorists have developed random utility maximization models and experimentalists routinely apply probabilistic choice models to fit their data, often without considering the source of the noise in the decisions [5–7]. One prominent conjecture is that individuals do not know their preferences, but instead must construct their preferences at the time of choice.

The promise of neuroeconomics has been to use knowledge from neuroscience and psychology to improve our models of economic decision making [8–19]. One obvious direction for improvement is to develop dynamical models that can jointly predict choices and reaction times (RT). RTs are routinely ignored in economics experiments, presumably because they are irrelevant in the static models that dominate the field. However, we know from the literature on sequential sampling models (SSM) that RTs can be used to more accurately predict choice behavior. So there is hope that dynamical models will prove to be a useful alternative to their static counterparts when RT data or other process measures are available.

Another direction for improvement is to develop models that can be applied in different domains. Currently we have separate models for risk aversion, temporal discounting, social preference, etc. Because these models take different inputs and result in arbitrarily scaled utilities, there has been little attempt to unify them. However, recent findings from neuroeconomics indicate that value coding in the brain is subject to adaptive coding. This suggests that the range of inputs to the decision mechanism in one task may be very similar to that in a different task, regardless of the actual range of values being considered. For example, an item with a subjective value rating of 5 out of 10 may be encoded at the same level as a $50 reward in a $0–100 experiment or 10 points in a 0–20 point experiment. Thus it may be possible to predict choice probabilities and RTs across tasks if the range of values is accounted for and exogenous factors like time pressure are not too different.

The model we use to explore these possibilities is a SSM closely related to the drift-diffusion model (DDM), which has received a great deal of attention for its ability to fit choices, RTs, eye-movements, and measures of neural activity during certain types of decision making [10,12,15,16,20–30]. While most applications of SSMs have been in the perceptual decision-making domain, they have also been applied to some value-based tasks [15,16,22–25,28,31]. Here we build off of this work and explore (a) whether a SSM can capture social decisions, and (b) whether the parameters fit to a binary food choice task can help predict behavior in four separate social-decision tasks.

It is important at the outset to address some unique aspects of this work. First, one might assume that individual variability must surely make our attempt to explain out-of-sample choice behavior an exercise in futility. While it is likely true that individual subjects will vary in terms of their parameters, those variations will average out in sufficiently large datasets. Many applications of behavioral models are concerned with aggregate behavior and that is our focus here as well. Accounting for individual variability in behavior is certainly a promising direction, but it is not the aim of this paper. Second, as alluded to above, we do not wish to claim that there are universal parameters that will precisely predict data in every new dataset. Rather we think of these as “baseline” parameters meant to capture the fact that, in the aggregate, experimental subjects will tend to put in similar levels of effort to make quick and accurate decisions, regardless of the task in front of them. Factors like time pressure, performance improvement or degradation over time, and experimental manipulations will certainly alter these parameters, but hopefully in predictable ways. Investigating such alterations is also separate from the aims of this paper.

Instead, a primary goal here is to address the criticism that SSMs involve too many free parameters and so could conceivably fit nearly any set of choice and RT data ([32], but see [20,33]). We address this criticism by estimating the parameters in one dataset and then assessing how well the model performs with these parameters in other datasets. Thus, this is not a demonstration of how well we can fit the model to any new dataset, but rather how well the model can perform out-of-sample with minimal or no fitting.

## Results

We show that a SSM fit to a food-choice experiment [15,16] is able to accurately predict choice and RT profiles in several different social-preference experiments. While these choice scenarios differ in terms of the types of rewards (food and money), the locations of the experiments (U.S.A. and Switzerland), and the choice environments (behavioral and functional magnetic resonance imaging (fMRI)), they also share important commonalities. In all cases, subjects were isolated and made self-paced computerized preference decisions. The social decisions were all anonymous one-shot interactions, thus minimizing reputational concerns. Furthermore, value-based choice studies, including food and social decisions, typically show activation of a common network of brain regions including the ventromedial prefrontal cortex and striatum [24,34–38]. Thus despite the apparent differences between tasks, we have reason to believe that they share a common decision mechanism.

### Task descriptions

**Fig 1:**
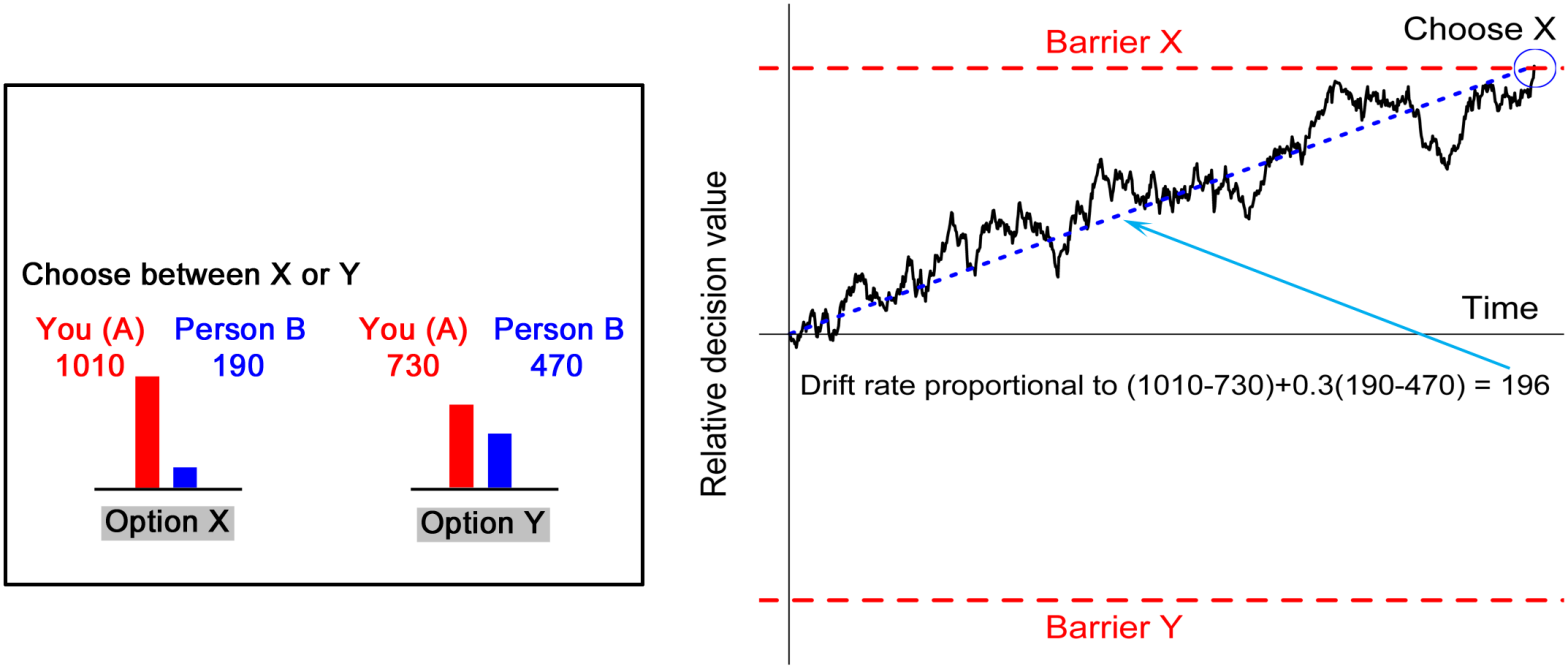
An example of a decision from Task 1, with selfish option X on the left and the fairer option Y on the right along with a simulated decision for this example, using the social aDDM with the parameters from the food study. The drift rate (dotted blue line) is proportional to the weighted sum of the payoff differences for each player between options X and Y. The weight of 0.3 is the discount parameter θ.

Task 1 provides behavioral data from a task using the Dictator game where 30 subjects in the role of the dictator made 70 binary decisions between two allocations of money, each one specifying an amount for the dictator and an amount for the receiver (Fig 1). For each choice there was a “selfish” option and a “fairer” option. Compared to the fairer option, the selfish option gave more money to the dictator and less money to the receiver (see Methods).

Task 2 is a behavioral task consisting of 69 subjects who faced 100 different binary choice problems. These decisions again involved choosing between two allocations of money, but this time there was, apart from the dictator and the receiver, a third subject involved (a “co-dictator”) who always received the same amount as the dictator. As in Task 1, the selfish option always resulted in more money for the dictators and less money for the receiver. An important feature of Task 2 is that while the game consists of three players, there are only two unique sets of payoffs for the dictator to consider. Existing models of social preference would predict that there should be more weight put on the dictator’s payoff compared to the standard Dictator game from Task 1. For example, the widely-used Fehr-Schmidt model predicts that with the extra partner, the weight on the receiver’s payoff should decrease by half [39]. However, as detailed below, our model predicts no change from Task 1. This is because the dictator and co-dictator’s payoffs are identical and we assume that consistently redundant information is ignored in the choice process. So as in Task 1 there are only four payoffs to consider, two for the dictators and two for the receiver.

Task 3 (n = 30) was identical to Task 2, except that in Task 3 we removed the redundant column of information so that there were only four payoffs on the screen, two for the dictators and two for the receiver. We anticipated no difference in behavior between Tasks 2 and 3.

Task 4 involves the behavioral data from a previously published fMRI study on the Ultimatum game [36]. In the Ultimatum game, one subject (the proposer) offers some division of a fixed amount of money to another subject (the responder). The responder can either accept the offer, or he can reject the offer, leaving both players with nothing. In this game, subjects often reject unfair offers, contrary to economic models assuming pure self-interest. Rejection behavior in these games directly implies that receivers of unfair offers have a negative valuation of the proposer’s payoff. In this study, 18 subjects were asked to respond to 16 Ultimatum offers, each from a total pie of 20 Swiss Francs (~$25), and subjects received offers of 4, 6, 8, and 10 Swiss Francs.

### Computational model

In a SSM, there exists a latent variable, which we have termed the relative decision value (RDV). Others have referred to this variable simply as “information” or “net evidence”. In our version of the model, the RDV begins each new decision with a value of zero. As the decision maker accumulates evidence, the RDV evolves until it reaches a threshold value of +/- *b*, at which point he/she chooses the corresponding option. For each step in time (here 1 ms.), the RDV changes by an amount equal to the drift rate plus Gaussian white noise with standard deviation σ. Because the RDV has arbitrary units, we are always free to set the drift rate, the barrier height (*b*), or the noise (σ) equal to 1. To allow comparison with our previous results on food choice, we fix the decision barriers to one (i.e. *b* = 1).

A critical question in applying SSMs to value-based decisions is how to handle the drift rate. The drift rate represents the average rate of net evidence accumulation for one option over the other. In a standard perceptual experiment, there would be several conditions with different levels of discriminability. A separate drift rate would be fit to each condition and they would reflect the strength of the perceptual evidence, i.e. the task difficulty. In value-based decision making, discriminability is determined not by perceptual qualities of the items, but by the strength of preference for one item over the other. In a typical experiment every decision problem is unique so there can be a continuum of preference levels. In principle one could try to bin certain trials together and fit separate drift rates to different strengths of preference (indeed this is how we visualize the model fits). However, which trials “fit together” can depend on parameters of the model and so the modeler may be forced to make arbitrary decisions about how to bin the data. Moreover, this approach discards valuable information about how changes in preference lead to changes in drift rate.

Our approach is to instead let drift rate vary continuously as a function of the strength of preference. This way we fit all of the data at once, using just one drift parameter *d*, which multiplies the underlying value difference between the two options. For example, a choice between a $1 item and a $2 item would have a drift rate of *d*, while a choice between a $1 item and a $5 item would have a drift rate of 4*d*. More concretely, our model can be written as follows:

$$V_{t}=V_{t-1}+d\left(x-y\right)+\varepsilon_{t}$$

where *V* is the RDV, *x* and *y* are the underlying values for option X and option Y respectively, and ε is Gaussian noise with mean zero and variance σ^2^. *V* starts each trial at 0 and evolves over time until it reaches +/-1.

Recent food-choice experiments have used eye-tracking data to demonstrate that visual fixations play an important role in the evidence accumulation process [15,16]. These results led to the introduction of the attentional DDM (aDDM) where evidence is accumulated more quickly for an option when it is being looked at than when it is not. This effect is captured by a fixation-bias parameter θ that discounts the value of the unlooked-at option when calculating the current drift rate, and it leads to a substantial choice bias for options that happen to capture more looking-time over the course of a trial. The estimated bias parameter value in that study (which has been validated in multi-option choice in [16]) was θ = 0.3, meaning that the values of the unlooked-at food items are discounted by a factor of roughly a third during comparison.

Here we hypothesize that, much like the unlooked-at food items, other peoples’ payoffs receive less weight than one’s own payoffs during the choice process. When subjects make choices in an experiment that involves their own monetary payoff and another player’s monetary payoff, they have to compare these payoffs across alternatives to make a decision. For example, if subject *i* earns *x*_*i*_ in alternative X and *y*_*i*_ in alternative Y the subject needs to compare these values. Likewise, the subject needs to compare the monetary payoffs *x*_*j*_ and *y*_*j*_ for subject *j* in the two alternatives. We can parsimoniously capture these comparisons in an extended DDM as follows:

$$V_{t}=V_{t-1}+d^{s}\left[\left(x_{i}-y_{i}\right)\pm \theta^{s}\left(x_{j}-y_{j}\right)\right]+\varepsilon_{t}$$

In this model *d* ^*s*^ denotes the drift-rate multiplier and *θ* ^*s*^ measures the subject’s discount rate for the other player’s payoff. This aspect of the model is inspired by Decision Field Theory, where attentional weights dictate the influence of different attributes on the decision [23,40]. Note, that the discounted payoff difference *θ* ^s^(*x*_*j*_−*y*_*j*_) for the other player may enter the relative decision value either positively or negatively depending on how the subject values the other player’s payoff.

In principle, it is possible to estimate the drift rate *d* ^*s*^, the standard deviation of the error term σ^s^, and the discount rate *θ* ^*s*^ for each of our four social-preference experiments. Instead, we would like to pose a different question. Using parameters fit to one task (a food-choice experiment), how well can the aDDM capture choices and RTs in separate, random groups of subjects in social-decision experiments? Because the social-decision datasets do not include eye-tracking data, we need to make a few extensions to the model.

First, we assume (based on [16]) that on average subjects allocate equal attention to both choice options. Second, we assume that consistently redundant information is ignored in the choice process (we test this assumption in Tasks 2 & 3). Third, we incorporate adaptive coding [41,42] to account for the fact that each experiment has different units and ranges of value (see Methods). Finally, we assign a weight of θ = 0.3 to the other subject’s payoff. There are a couple of motivations for this choice. Initially we hypothesized that subjects in these tasks would be focused on themselves and thus discount the other subjects’ payoffs in the same way that unattended items are discounted in the food-choice aDDM. We later confirmed this hypothesis with *ex-post* model fits of the θ parameter to each dataset. However, we cannot rule out that this may be a coincidence.

In order to capture the stochastic nature of the aDDM we simulate the model 1000 times for each choice problem presented to the subjects and create 95% confidence intervals using bootstrapped samples equal in size to the actual datasets. We then compare the simulated choice and RT curves to the real data. Note that the inputs to these simulations are only the monetary values of the options X and Y for each trial (e.g. Fig 1). No data regarding the subjects’ choices or RTs in the social-decision tasks at the individual or group levels were used to generate predictions for these tasks.

### Model predictions

**Fig 2:**
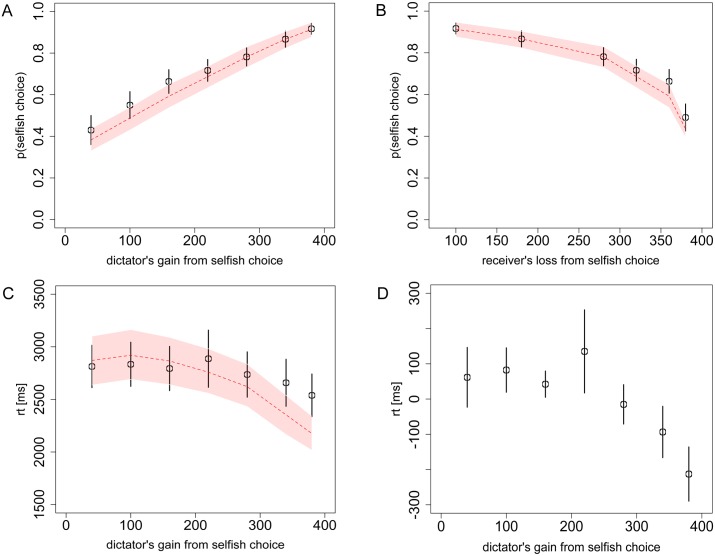
Choices and reaction times from Task 1 (Dictator game). **A)** The probability of the dictator choosing the selfish option as a function of his own payoff gain from the selfish option, relative to the fairer option, and **B)** as a function of the receiver’s payoff loss from the selfish option. **C)** The dictator’s RT, and **D)** individually de-meaned RT, as a function of his own payoff gain from the selfish option. Black circles indicate the aggregate subject data with bars representing s.e.m. Red dashed lines indicate the aDDM predictions with 95% confidence intervals. Overlap of these confidence intervals with the data’s standard error bars in every case indicates excellent model predictions. In the first row of the figure (and Figs 3 and 4) the two panels present the same data but using different x-axes to demonstrate that the data and model are sensitive to both dimensions of the decision problem.

**Fig 3:**
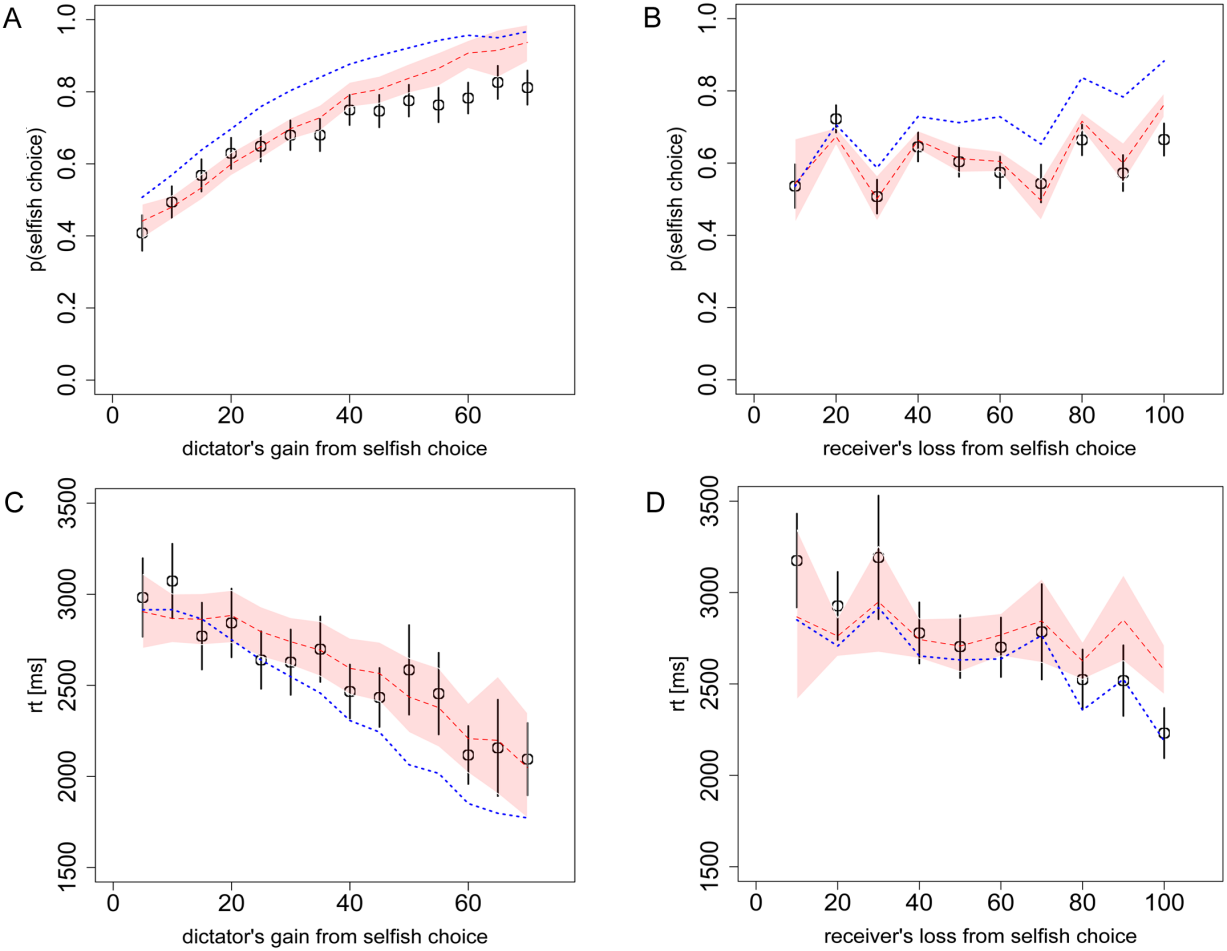
Choices and reaction times from Task 2 (Dictator game). **A)** The probability of the dictator choosing the selfish option as a function of his own payoff gain from the selfish option, relative to the fairer option, and **B)** as a function of the receiver’s loss from the selfish option. **C)** The dictator’s RT as a function of his own payoff gain from the selfish option, and **D)** as a function of the receiver’s loss from the selfish option. Black circles indicate the aggregate subject data with bars representing s.e.m. Red dashed lines indicate the DDM predictions with 95% confidence intervals. The overlap of these confidence intervals with the data’s standard error bars indicates excellent model predictions. Blue dotted lines indicate the alternative DDM with *θ* = 0.15, which would be the prediction from the widely-used Fehr-Schmidt model of social preferences.

**Fig 4:**
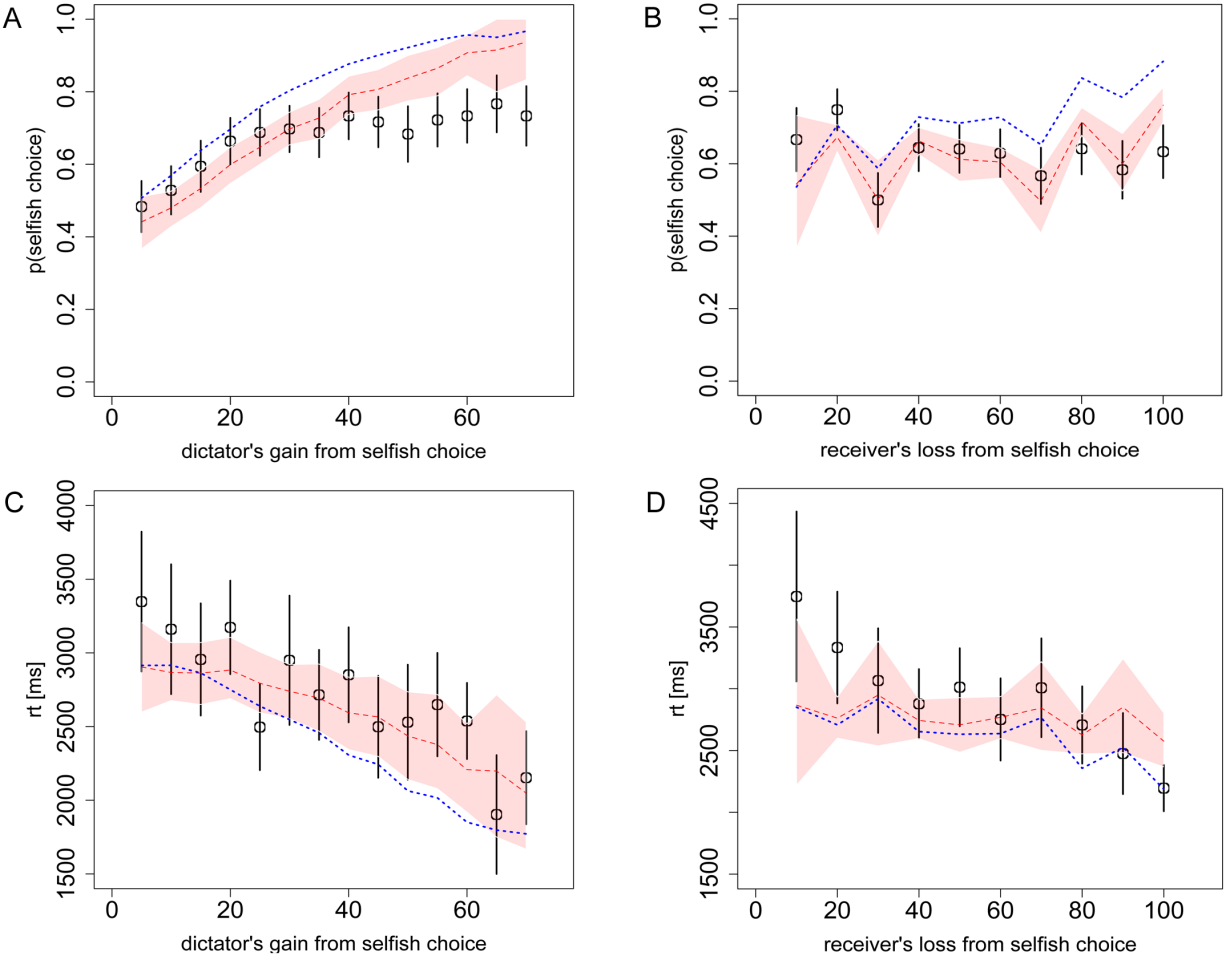
Choices and reaction times from Task 3 (Dictator game). **A)** The probability of the dictator choosing the selfish option as a function of his own payoff gain from the selfish option, relative to the fairer option, and **B)** as a function of the receiver’s loss from the selfish option. **C)** The dictator’s RT as a function of his own payoff gain from the selfish option, and **D)** as a function of the receiver’s loss from the selfish option. Black circles indicate the aggregate subject data with bars representing s.e.m. Red dashed lines indicate the DDM predictions with 95% confidence intervals. The overlap of these confidence intervals with the data’s standard error bars indicates excellent model predictions. Blue dotted lines indicate the alternative DDM with *θ* = 0.15, which would be the prediction from the widely-used Fehr-Schmidt model of social preferences.

**Fig 5:**
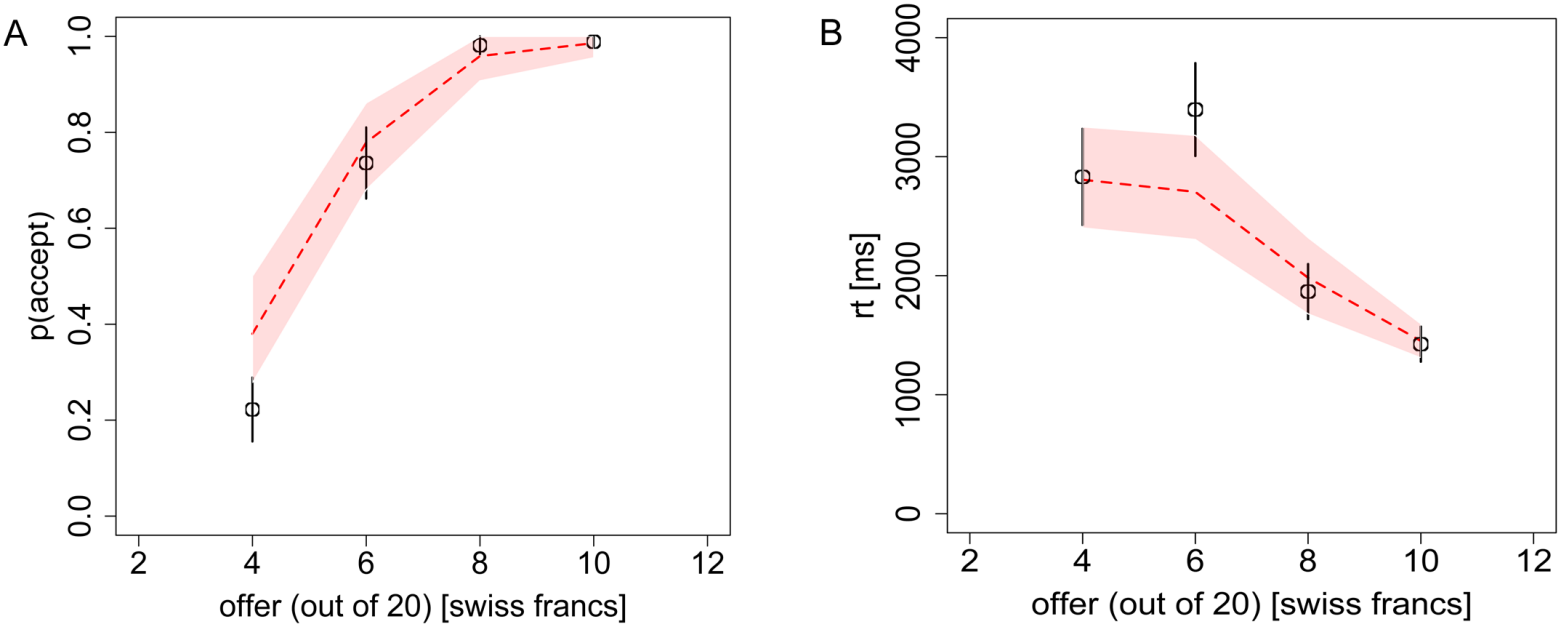
Choices and reaction times from Task 4 (Ultimatum game). **A)** The probability of the receiver accepting an ultimatum offer, and **B)** the RT, as a function of the offer (out of a possible 20 Swiss Francs). Black circles indicate the aggregate subject data with bars representing s.e.m. Red dashed lines indicate the DDM predictions with 95% confidence intervals. Overlap of these confidence intervals with the data’s standard error bars in every case, indicates excellent model predictions.

Figs 2–5 show the predicted choice and RT curves from the aDDM and the actual data. The model does a remarkable job of predicting both the shape and the absolute levels of the choice probability and RT curves in each of the four tasks, as evidenced by the consistent overlap between the 95% confidence intervals of the model and standard error bars of the data. In S1 Fig we also show that the model accurately predicts the entire RT distributions for each dataset [20].

In addition to the figures, the predictive accuracy of the aDDM can also be assessed in quantitative terms. To do so we calculate the mean error magnitudes between the model and the data (Table 1) as well as traditional goodness-of-fit tests for all four tasks (see S1 Table).

**Table 1:** Model error rates. As described in the results, we assessed each model’s prediction accuracy by calculating the mean absolute difference between the model prediction and the observed data across the bins in Figs 2–5. For each set of data (task + choice/RT) the most accurate model is indicated in bold. The asterisk in the first row indicates that the Bolton-Ockenfels model was actually fit to the choice data from Task 1, so it is not surprising that it provides the best fit.

Error rates	aDDM	Fehr-Schmidt	Bolton-Ockenfels	Ornstein-Uhlenbeck
**Task 1 choice**	2.9%	4.2%	1.7%*	12.9%
**Task 1 RT**	**5.9%**	15.9%	7.7%	53.4%
**Task 2 choice**	**4.5%**	8.0%	16.6%	13.9%
**Task 2 RT**	4.2%	**3.8%**	9.5%	49.1%
**Task 3 choice**	**7.4%**	8.8%	17.3%	14.8%
**Task 3 RT**	**7.3%**	7.7%	12.9%	47.7%
**Task 4 choice**	**5.7%**	15.1%	21.2%	17.3%
**Task 4 RT**	**6.3%**	15.3%	20.2%	36.2%
**Mean choice**	**5.1%**	9.0%	14.2%	14.7%
**Mean RT**	**5.9%**	10.7%	12.6%	46.6%
**Overall mean**	**5.5%**	9.9%	13.4%	30.7%

In Task 1 (Fig 2A-2B) we see that the probability of choosing the selfish option generally tends to increase with the dictator’s own payoff gain (compared to the other option), and to decrease with the receiver’s payoff loss from the dictator’s selfish option. In other words, while dictators are more likely to choose the selfish option if it earns them more money, they are less likely to choose an option that reduces the receiver’s earnings. Looking at the RTs, we see that the quickest decisions generally occur when the selfish option leads to the most money for the dictator and to the smallest loss for the receiver (Fig 2C-2D and S2 Fig). This decrease in RT is about a third of a second or 12% compared to the slowest decisions. Furthermore, in a mixed-effects regression of log(RT) on |p-0.5|, where p is the mean group probability of choosing the selfish option, we find a highly significant effect (p = 0.0005).

The average error magnitudes between the model and the data (as a function of the dictator’s payoff gain and the receiver’s payoff loss, respectively) are only 3.1% and 2.7% for the choice curves (Fig 2A–2B) and 5.6% and 6.1% (of the highest mean RT) for the RT curves (Fig 2C and S2 Fig).

In Task 2 we hypothesized that adding the co-dictator without adding additional payoffs to consider would not increase the dictator’s focus on that payoff and thus would not change the relative weight between the dictators and the receiver. In other words we predicted that the dictator would treat the decision problem as if there were only two players: the dictator and the receiver. Contrast this with an alternative scenario where the dictator’s and co-dictator’s payoffs are different; in that case the co-dictator’s payoff would receive a weight of 0.3, just like the receiver’s payoff. Note that in the extreme, the model makes the same prediction for any number of co-dictator or receiver subjects, as long as there are not additional different payoffs to consider. While counterintuitive, this “scope insensitivity” effect has been well documented in the literature on contingent valuation [43].

As expected, in Task 2 (Fig 3) the probability of choosing the selfish option steadily increases with the dictators’ payoff gain, while there is no linear trend as a function of the receiver’s payoff loss. In this task there was systematic variation in the size of the tradeoff between money for the dictator and money for the receiver. For example, trials with losses to the receiver of 10 and 30 had dictator gains of 5 and 10, while trials with losses to the receiver of 20 and 40 had dictator gains of 17 and 23. Thus the selfish option was more appealing in the latter trials and was more likely to be chosen, as seen in Fig 3B. So, what initially looks like random noise in Fig 3B is actually due to systematic variations in the dictator’s gains and is well-predicted by the model (mean error magnitude: 5.6% for the dictator’s gain (Fig 3A), 3.4% for receiver’s loss (Fig 3B)). Fig 3 also shows that RTs increase as both the dictators’ and the receiver’s payoff differences between the two options go to zero (i.e. for the most similar options) and that the model captures these trends well (mean error magnitude: 3.1% for the dictator’s payoff gain (Fig 3C), 5.2% for receiver’s loss (Fig 3D)). The correspondence between predicted and empirically measured RT is also high when using the model to combine dictator and receiver payoffs into a single utility measure (S2 Fig).

In Fig 3 we also present simulations of the model with *θ* = 0.15 to demonstrate how the model misfits the data if we assume that the receiver’s payoff receives only half of the weight from the normal Dictator game (Task 1), as would be the case, for example, in the Fehr-Schmidt model [39]. Below, we present data on explicit tests of the Fehr-Schmidt model.

As expected, behavior in Task 3 closely matched behavior in Task 2. Overall subjects chose the selfish option on 64% of trials in Task 2 and 65% of trials in Task 3 (t = 0.173, p = 0.86). At a more detailed level, we observed similar choice and RT curves (Fig 4 and S2 Fig) (choice: mean error magnitude: 9.1% for the dictator’s gain (Fig 4A), 5.6% for receiver’s loss (Fig 4B), RT: mean error magnitude: 6.5% for the dictator’s gain (Fig 4C), 8.1% for receiver’s loss (Fig 4D)).

In Task 4 we observe the standard behavioral trend that subjects almost always accept “fair” or “almost fair” offers of 40–50% of the total pie, but start to reject lower offers (Fig 5), with an average indifference point in the 20–30% offer range. Again, the model provides accurate predictions for both choices (mean error magnitude: 5.7%) and RT (mean error magnitude: 6.3%).

### Alternative models and parameters

#### Alternative θ values

To test our assumption of θ = 0.3 in all four datasets, we can ask precisely how much the other player’s payoff is discounted (relative to the subject’s own payoff) in each of the four social-preference experiments. For this purpose, we performed *ex-post* maximum likelihood estimates for the θ parameter in each social-preference dataset. Finding similar focus parameters in each of the four independent datasets would provide another piece of evidence consistent with a common underlying choice mechanism. These tests yielded best-fitting focus parameters of 0.21, 0.32, 0.33 and 0.31 for the four tasks respectively, and in Task 1 we could not reject (using a likelihood-ratio test) that the parameter falls in the interval [0.25,0.35] from the previous food choice study (see S3 Fig). Also, the average differences (across choice and RT figures) between the best-fitting model and the model with *θ* = 0.3 were just 0.75%, 0.29%, 0.15% and 0.63% for Tasks 1–4 respectively. In S4 Fig we show examples where model predictions using *θ* = 0.2 and *θ* = 0.4 yield clearly worse fits to the data. These results are consistent with the idea that the θ parameter is stable across datasets.

**Fig 6:**
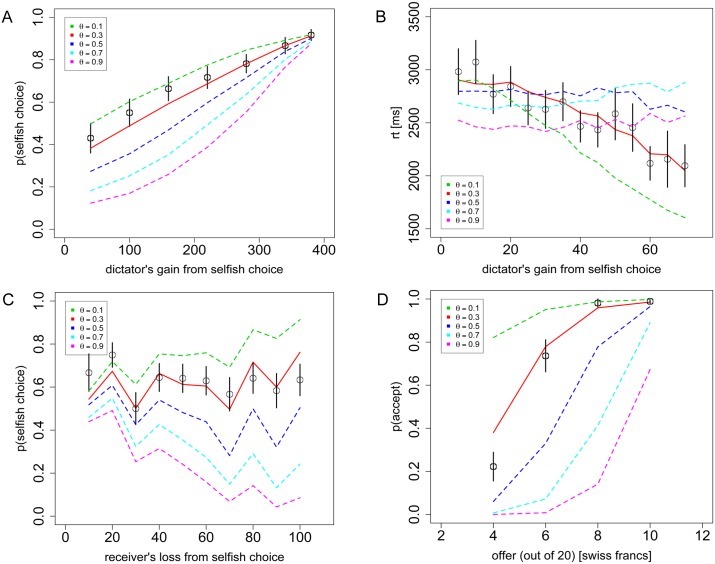
Sensitivity of the model to the parameter θ. **A)** Replication of Fig 2A, **B)** Fig 3C, **C)** Fig 4B, and **D)** Fig 5A, using θ = {0.1, 0.3, 0.5, 0.7, 0.9}. Black circles indicate the aggregate subject data with bars representing s.e.m. Dashed lines indicate the aDDM predictions with different θ values, while the solid red line indicates the predicted θ = 0.3.

To further illustrate the importance of the parameter θ, in Fig 6 we display the range of choice and RT curves possible with θ = {0.1, 0.3, 0.5, 0.7, 0.9}, holding the other parameters constant. These curves highlight a key feature of the model, which is the relationship between the social allocations and evidence accumulation or drift rate. For any given choice problem, the value of theta can drastically affect the drift rate and thus the resulting choice-probability and RT distributions. Without this feature of the model, there would be no clear way to predict behavior across trials.

#### Alternative social preference models

**Fig 7:**
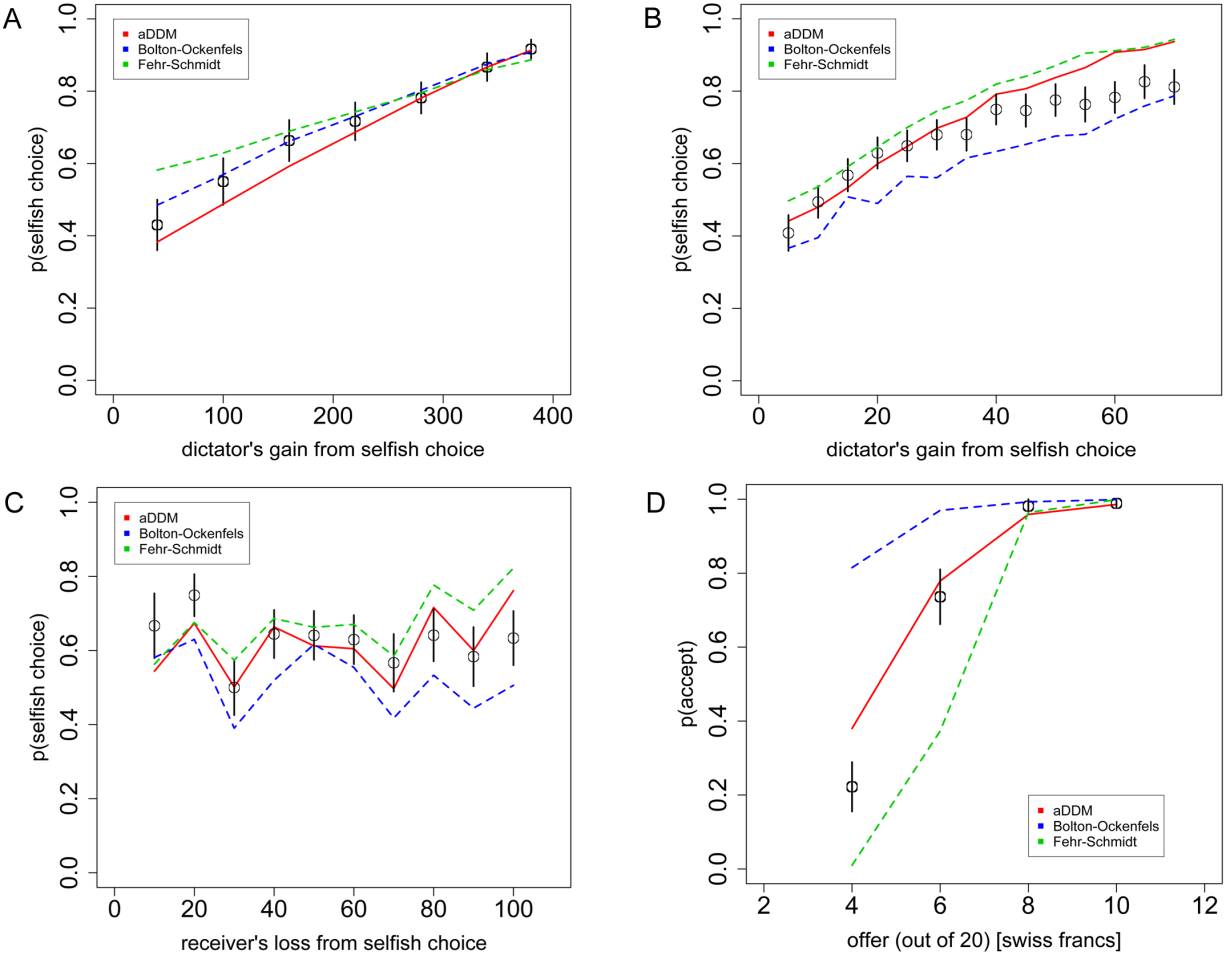
Alternative social preference models. **A)** Replication of Fig 2A, **B)** Fig 3A, **C)** Fig 4B, and **D)** Fig 5A, using the alternative Fehr-Schmidt and Bolton-Ockenfels models, as well as the aDDM. Black circles indicate the aggregate subject data with bars representing s.e.m. Dashed lines indicate the alternative model predictions, while the solid line indicates the aDDM prediction.

To determine whether other social preference models might fit the choice data as well as our proposed model, we tested two prominent social-preference models, the Fehr-Schmidt and Bolton-Ockenfels models (see Methods). For the Fehr-Schmidt model we took parameter values reported in the original article [39]. For the Bolton-Ockenfels model, no parameter values are reported in the original text [44], and we are not aware of any accepted values in the literature. Therefore, to test the Bolton-Ockenfels model we fit the model to the choice data from Task 1 and then predicted on Tasks 2–4. Fig 7 shows the predicted choice curves from Tasks 1–4 for each of the social preference models.

These alternative models capture the general patterns of choice behavior in the Dictator games (particularly the Bolton-Ockenfels model on Task 1 to which it was fit), but they perform considerably worse on the Ultimatum game (Task 4). Moreover, it is important to note that, in their standard form, none of these models make any predictions about RT, which is why we are focused on choice predictions. Nevertheless, when we implement these models within a SSM framework, they can produce RT predictions, which we present in S5 Fig. Note that the SSM implementations of the social-preference models also generate probabilistic choice curves, which we use in lieu of standard logistic/softmax choice functions to produce the Fig 7 plots. Error rates for these alternative models are presented in Table 1. Overall the average error rates for the Fehr-Schmidt and Bolton-Ockenfels models were 9.9% and 13.4%, compared to just 5.5% for the aDDM.

#### Alternative sequential sampling models

**Fig 8:**
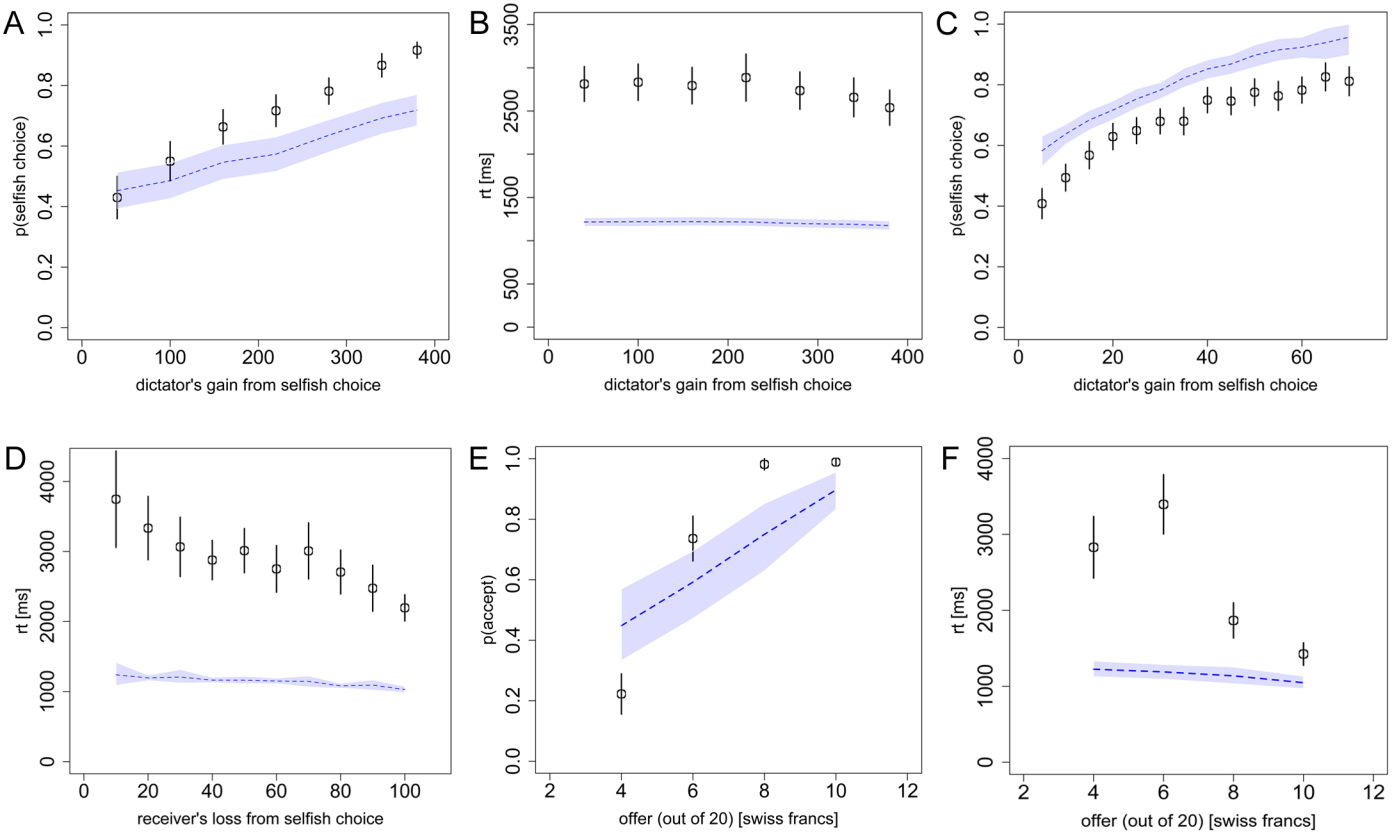
Alternative sequential sampling model. **A)** Replication of Fig 2A, **B)** Fig 2B, **C)** Fig 3A, and **D)** Fig 4C, **E)** Fig 5A, and **F)** Fig 5B, using the alternative Ornstein-Uhlenbeck model. Black circles indicate the aggregate subject data with bars representing s.e.m. Dashed lines indicate the alternative model predictions with 95% confidence intervals.

Given the demonstrated importance of the social-preference function and the focus parameter *θ*, it is natural to ask whether the particular SSM and parameters are also important, or whether any SSM formulation might capture the choice and RT patterns equally well. To answer this question we tested an alternative SSM, the Ornstein-Uhlenbeck (OU) model [45], using parameters values that were also originally fit to a food-choice study [46]. The OU model differs from the aDDM in that it allows the accumulation process (i.e. drift rate) to vary as a function of the RDV, either increasing or decreasing as the RDV nears the decision boundary. For the inputs to the OU model, we used the same weighted sum of self and other payoffs as in the aDDM. Fig 8 shows predicted choice and RT curves from Tasks 1–4 using this model.

While this model does predict similar qualitative relationships between choice, RT and the payoffs, the accuracies of those predictions are substantially worse than the aDDM, particularly for RTs (average error rate = 30.7%; see Table 1). This demonstrates that in addition to requiring the proper social-preference model, it is also important to use an appropriate SSM implementation of those social preferences.

## Discussion

Taken together, our results show that social decisions display relationships between choice probability and RT that are consistent with a dynamical decision process and that these relationships can be quantitatively captured with a sequential sampling model. Additionally, we demonstrated that these four particular datasets were accurately predicted by parameters from a previous food-choice model.

These results are consistent with the notion that there is a common computational framework by which value-based decisions are made. More broadly, the established success of SSMs in describing memory and perception suggests that these models are capturing fundamental aspects of decision making. Namely, a decision-maker evaluates options at the time of choice by accumulating evidence for them until one of the options is judged as being sufficiently better than the other(s). In perceptual decision making the stochastic evidence is primarily exogenous while in value-based decision-making the stochastic evidence is generated internally. Nevertheless, similar evidence-comparison processes seem to apply in these different cases.

It is important to highlight that the particular model we have used here is simplified in the sense that it does not include additional parameters for variability in drift rate or starting point across trials or subjects. One strength of SSMs is that they predict not just mean RT, but entire RT distributions. While our model captures these distributions reasonably well, additional parameters are likely required to fully capture every facet of the data. While our model may sacrifice some amount of precision in these distributional analyses, we believe this is outweighed by the benefits of simplicity and robustness across different choice environments.

The benefits of this robustness are apparent from our ability to predict behavior across the different datasets. Of course, factors like time pressure, performance improvement or degradation over time, and experimental manipulations will likely alter the parameters of the model. It also remains an open question whether these same parameters will predict behavior outside of the lab where focus and its discounting effects on choice can be affected by many uncontrolled factors such as distraction and advertising. For instance, we know already from a large body of work that putting subjects under time pressure causes faster and less accurate decisions, reflected by a change in parameters of the SSM (e.g. [22,46,47]). This means that if one cannot directly control for time pressure in the field environment, the parameters measured in the laboratory may not predict field behavior accurately. Studying how environmental/experimental factors influence these parameters is an important line of investigation to see how far we can stretch these models.

It is also important to highlight that the particular model we have used here is just one of the many SSMs in the literature, including the drift-diffusion model, linear ballistic accumulator model, leaky competing accumulator model, Ornstein-Uhlenbeck model, biophysical attractor models, and urgency-gating models [12,27,45,46,48–50]. While there is a good deal of interest and work being done to distinguish between these models, many of them are identical under certain parameter restrictions and otherwise tend to yield only subtle differences in RT distributions [45,51,52]. In other cases, the data themselves must have specific characteristics to allow comparisons between different SSMs. For example, it has been reported that the urgency-gating model is identical to the drift-diffusion model in situations with constant evidence, as in the datasets we test here [50]. Thus it is impossible to distinguish between the urgency-gating model and the aDDM in this setting. However, it is worth noting that the urgency-gating model would presumably not predict the effects of attention on the weighting of self and other payoff, and thus would be equivalent to the aDDM with *θ* = 1. As can be seen from Fig 6, that model would clearly fail to capture our subjects’ behavior.

In principle it is possible to test specific features of different SSMs against each other in value-based tasks, but to do so would likely require considerably more data, as these sorts of model-fitting exercises are typically performed with ~1000 trials per subject, whereas here our tasks ranged from 16 to 100 choices per subject, spread over a continuum of “conditions” (i.e. subjective-value differences).

Even with smaller datasets like the ones reported here, the close relationship between choice probabilities and RT provides additional possibilities to test and constrain the models. We tested several alternative models, both in terms of the subjective value function and in terms of the SSM framework. Specifically we compared our aDDM with Fehr-Schmidt and Bolton-Ockenfels social-preference models and an Ornstein-Uhlenbeck SSM [39,44,46]. While each of these models adequately captured some aspects of some tasks, none were able to match the predictions of the aDDM across tasks and measures (choice probability and RT). Furthermore, the Fehr-Schmidt and Bolton-Ockenfels models do not predict RT at all without inserting them into a SSM such as our aDDM.

RTs have been woefully understudied in economics, potentially because of a perceived lack of formal models that make use of them. SSMs provide a clear and precise link between RT and the choice problem. Longer RTs are a direct result of smaller differences in subjective value between choice options, with the longest RTs coinciding with a subject’s indifference point (see S2 Fig). This follows from the fact that smaller value differences lead to slower net evidence accumulation and consequently a higher latency to cross a choice threshold. The measurement of indifference points is the basis for inferring preferences and our model implies that using RTs can improve the estimation of preferences, which should be of great interest to the field. Furthermore, the fact that people consistently spend the most time on decisions that matter the least, i.e. where the subjective value difference between the choice options is smallest, has important normative implications for economics [15,22,25,28,31,53] (but see [54,55]for a more nuanced discussion of this issue).

The proposed role of focusing effects in social decisions suggests novel interventions, namely the possibility of changing subjects’ behaviorally revealed social preferences by guiding their focus to other players’ payoffs. Such experimental manipulations have been shown to alter choice patterns (e.g. [56]). This possibility stands in sharp contrast to the orthodox view in economics that takes preferences as given and unchangeable [57]. It also stands in contrast to the prevailing social preference models in economics (e.g. [39,44,58,59]) that neglect the focusing effects underlying other-regarding behaviors. There is indeed evidence suggesting that fixations to other subjects’ payoffs is predictive of other-regarding behavior [60], which provides support for our approach. Interestingly, these results are also consistent with findings from individuals with autism and amygdala damage [61,62], who are impaired both in making eye contact and in social behavior. Based on these findings, it has been argued that making eye contact is a way of directing focus to social, rather than selfish outcomes. While manipulating their attention has been successful in improving the social behavior of autistic subjects, it has yet to be shown that such manipulations can alter social preferences in normal subjects. However, manipulating social distance through the wording of the instructions or identity of the subjects has been shown to change social behavior, possibly by focusing subjects’ more on others’ outcomes [63,64]. The SSM framework, as a general model of decision making, provides a means of quantifying the effects of such manipulations on choice behavior.

## Methods

### Ethics statement

Subjects gave informed written consent before participating in each of the four studies. All studies were approved by the local ethics committee (Zurich, Switzerland; KEK-ZH 2010–0327).

### Model details

There are four parameters that are standard in all DDMs: the standard deviation of the Gaussian noise (σ), the drift parameter (*d)*, the decision barrier height, and a non-decision time. Because the relative decision value has arbitrary units, we are always free to set one of the first three parameters in the DDM equal to 1. Following the convention from our previous papers, we fix the decision barriers at +/- 1.

The first parameter (σ) dictates the amount of noise added to the average drift rate at each millisecond time step, and its value is taken directly from the food-choice experiments (σ = 0.02).

The second parameter is the drift parameter *d* that multiplies the value difference between the options, in order to determine the average drift rate. Because our formulation of the DDM has fixed decision barriers at +/- 1, the *d* parameter must be adjusted to the range of values in order to ensure the equivalence of the model across experiments with different units and/or ranges of value. For example, if in a certain experiment the payoffs are expressed in dollars rather than in cents, the nominal payoff range in the experiment with the dollar representation is smaller by a factor of 100 although the real payoffs are identical. It is therefore necessary to adjust the drift parameter *d* by a factor of 100 because otherwise the model would make wildly different predictions for two identical decisions. Similarly, a dollar difference in value has very different consequences if we are choosing between snack foods or luxury cars [41,42]. Thus, if different experiments involve different payoff ranges there is the need to adjust the drift parameter such that the product of the drift parameter *d* and the payoff range $\bar{v}$ remains constant across experiments. Note, that because $d\cdot \bar{v}$ is constant across all experiments this does not introduce any degree of freedom in choosing the value of *d* in the social-preference experiments examined here.

In the social-preference experiments the subjects made choices sequentially and thus could not know the precise range of values that they would face. However, subjects could immediately see the order of magnitude of their payoffs, so we set the value of *d* such that the product of *d* and the order of magnitude of $\bar{v}$ is always equal to 0.002 ms^-1^, the value from the food choice studies. See S2 Table for details.

From the binary food-choice study we know that fixation durations do not depend on the values of the options so we can assume that, on average, each option is focused on for half of the trial. Therefore, each option (e.g. option X) accumulates evidence at a rate of *d*·*x* for half of the trial, and 0.3·*d*·*x* for the other half of the trial, meaning that the average evidence accumulation rate for each option is [0.5·*d*·*x* + 0.5·0.3·*d*·*x*] = 0.65·*d*·*x*. In the context of the food-choice model this means that the average relative drift rate is equal to 0.65·*d*·(*x*−*y*). Again, we apply the behavioral insights from the food-choice study (i.e. that fixations do not depend on the values of the options) to the social-preference studies by assuming that each option is fixated on for half of the trial. Therefore, the final parameterized social DDM can be written as:

$$V_{t}=V_{t-1}+0.65d\left[\left(x_{i}-y_{i}\right)\pm 0.3\left(x_{j}-y_{j}\right)\right]+\varepsilon$$

where *x* and *y* are the amounts received by the decision maker (subscript *i*) and the other player (subscript *j*) for options X and Y. As before, the RDV evolves over time (in increments of 1 ms) until it reaches a value of +1 or -1. Note that in this extended version of the model, the factor of 0.3 appears twice, once to maintain the discount on the non-fixated choice options, and a second time to maintain the asymmetry between self and other payoffs. Another way to implement the model would have been to omit the asymmetry between self and other for the non-fixated option, but this alternative model provided worse fits to every dataset.

In our previous work [15] the non-decision time was not fit to the data, but instead directly measured from the food-choice data by calculating the average difference between RT and total fixation time. This difference was 355ms. In our simulations of the model we add this “non-decision time” to all the simulated RTs.

### Experimental procedures

#### Task 1 procedure

The Task 1 behavioral data is from an fMRI experiment conducted at the University of Zurich’s Social and Neural Systems laboratory. Subjects (n = 30) were asked to make a choice between two possible allocations of money, option X and option Y, where there was a tradeoff between their own payoff and the receiver’s payoff. The choice problems were presented in a random sequence, and the subjects were asked to make their decisions within 10 seconds. Subjects first observed a screen that announced the upcoming dictator game (“Choose X or Y”), followed by a decision screen that included the options X and Y. After the subjects made their choice using a two-button MRI-compatible button box, a fixation-cross appeared in the center of the screen during the inter-trial interval. If the subject did not make a decision within 10 seconds, then a choice was automatically made for the fair option and the experiment moved forward to the inter-trial interval. Three trials were excluded for failing to fall within this time limit. Inter-trial intervals varied between 3–7 seconds. Subjects also completed 50 additional trials that did not include a tradeoff between self and other payoff and so we do not analyze those trials here.

Prior to scanning, subjects read written instructions describing the task and the payoff rules. Comprehension of the payoff rules and the treatment conditions was tested by means of a control questionnaire. All subjects answered the control questions correctly and thus knew that they played with anonymous human interaction partners and that their decisions were treated in an anonymous way. The overall payment to the participants consisted of a fixed show-up fee of 25 Swiss Francs (CHF) plus the payment from six randomly selected choice problems. On average, participants earned 65 CHF (ranging from 55–79 CHF).

#### Task 2 procedure

In this experiment 69 subjects were run in two sessions at the University of Zurich's experimental economics laboratory. In the first session, the experiment consisted of two blocks, each with 100 choices. In each trial, subjects (n = 36) were randomly assigned into groups of three. Two subjects in the group (Players A & B) were “co-dictators” while the third subject (Player C) was a standard dictator-game receiver. In one block, subjects played the role of Player A, who could choose between two allocations of money (one fairer and one selfish) or could choose to delegate the decision to Player B. In the other block, subjects played the role of Player B, and were asked to choose between the same two allocations of money, assuming that Player A had delegated the decision to them. The order of the blocks was counterbalanced across subjects.

In this paper we only consider the Player B decision block, since those are the only binary decisions. Furthermore, we excluded the first trial from the 18 subjects who had the Player B decision block first, due to a software error in recording RTs.

Importantly, Players A and B always earned the same amount of money in every allocation, while Player C earned weakly less. Therefore, subjects only had to consider two payoffs for each allocation (four payoffs total), as in the other studies.

The payoffs for Players A, B and C were displayed in three separate columns, with the fairer option always above the unfair option. Subjects made their choices by clicking a radio button to the right of the option they wanted and then clicking an “ok” button at the bottom of the screen. There was then a variable waiting period until the last subject in the room entered their response.

At the end of the experiment, subjects were paid for two randomly chosen decisions. For each decision, 12 subjects were randomly assigned to the role of Player A, 12 to the role of Player B, and 12 to the role of Player C. Triads were formed and for each triad one choice scenario was chosen and played out according to the subjects’ choices in their designated roles. If Player A chose to delegate the decision, then the allocation was determined according to Player B’s choice; otherwise the allocation was determined from Player A’s choice and Player B’s choice was irrelevant. The roles were assigned so that no subject was ever assigned to the role of Player C twice, thus ensuring that each subject had at least one payment round as a Dictator. Subjects allocated “points” which were then converted into Swiss Francs at a rate of 0.12 CHF/point.

Unfortunately, in this first session RTs were recorded with a resolution of 1s, a limitation of the Z-Tree software. Therefore, we ran a second session of the experiment using Psychtoolbox (http://psychtoolbox.org/). This session was identical to the first except for the following differences: (1) Subjects (n = 33) made their choices by pressing the up or down arrow keys on the keyboard. (2) The delegation aspect of the game was removed; thus all subjects were in the role of Player B and only made 100 decisions. (3) Subjects were paid for 3 randomly selected trials, once for their own decision, once as the partner Player A, and once as the receiver Player C. (4) There was a two-second fixation screen between each decision. (5) The experiment was conducted in English rather than German. (6) The exchange rate was 0.06 CHF/point. Additionally, we excluded one trial from one subject, for failing to respond within a minute. See S6 Fig for a sample screenshot.

#### Task 3 procedure

This study was also conducted using Psychtoolbox. This session was identical to the second session of Task 2 except for the following differences: (1) There were only two columns of payoffs on the screen, one for the dictators and one for the receiver. (2) There were 30 subjects in this study. See S7 Fig for a sample screenshot.

#### Task 4 procedure

The fourth dataset was collected as part of a previously published experiment on the effects of transcranial magnetic stimulation (TMS) on Ultimatum game rejection behavior [36]. These subjects were the control group that did not receive any stimulation, but made their decisions while undergoing fMRI at the University of Zurich’s Laboratory for Social and Neural Systems Research. None of these subjects had any previous experience with Ultimatum games.

Every subject received four offers of 4 CHF, four offers of 6 CHF, three offers of 8 CHF, and five offers of 10 CHF. The offers were presented in a random sequence, and there was no time limit. The distribution of offers was generated from a previous pilot experiment. The proposers in this pilot experiment were asked whether their offers could be used again in subsequent experiments, and if they agreed, then they were actually paid based on subjects’ choices in the fMRI experiment. Therefore, the responders in the Ultimatum game faced real offers from other subjects and their choices affected those other subjects’ payoffs.

Each subject received 60 CHF as a show-up fee in addition to the money earned in the Ultimatum game experiment. Subjects knew that 8 of the 16 trials would be randomly selected at the end of the experiment for payment.

### Alternative social preference models

To simulate each model we used the given utility function to calculate the subjective value for each trial’s choice options (X and Y), and then used the difference between those utilities to determine the model’s drift rate. The model can be written as:

$$V_{t}=V_{t-1}+d\left(U\left(X\right)-U\left(Y\right)\right)+\varepsilon_{t}$$

The remaining parameters in the model (*d*, σ and non-decision time) remained the same as in the aDDM.

#### Fehr-Schmidt model

For a given social allocation among *n* players (*x*_*i*_ = own payoff, *x*_*j*_ = others’ payoffs) the Fehr-Schmidt utility is given by:

$$U_{i}\left(X\right)=x_{i}-\frac{\alpha }{n-1}\underset{j\neq i}{\sum }\text{max}\left\{x_{j}-x_{i},0\right\}-\frac{\beta }{n-1}\underset{j\neq i}{\sum }\text{max}\left\{x_{i}-x_{j},0\right\}$$

where *α* and *β* are free parameters that capture how much the decision maker dislikes disadvantageous and advantageous inequality, respectively. The parameter values we used were *α* = 0.85 and *β* = 0.315, as reported in [39].

#### Bolton-Ockenfels model

For a given social allocation among *n* players (indexed by *k*; *x*_*i*_ = own payoff, *x*_*j*_ = others’ payoffs) the Bolton-Ockenfels utility is given by:

$$U_{i}\left(X\right)=x_{i}-\beta {\left(\frac{x_{i}}{\Sigma_{k=1}^{n}x_{k}}-\frac{1}{n}\right)}^{2}$$

where β is a free parameter that determines how much the decision maker dislikes inequality.

We are not aware of any accepted values for β in the literature, so instead we fit the utility function to the aggregate choice data from Task 1 and then used that β parameter to predict Tasks 2–4. The model was fit using a maximum likelihood procedure assuming a standard logit choice function with temperature parameter λ. The best-fitting parameter was β = 2001. For Tasks 2–3 and Task 4, the ranges of payoffs were different (as described earlier) and so β was adjusted to values of 200.1 and 20.01 respectively.

### Alternative sequential sampling model

To test alternative SSM implementations we utilized the Ornstein-Uhlenbeck model. For a given choice problem we assume that the input to the model is the difference in subjective values between options X and Y, denoted *I* = *U*(*X*)−*U*(*Y*), where *U*_*i*_(*X*) = *x*_*i*_ ± 0.3*x*_*j*_ as in the main aDDM. Then the Ornstein-Uhlenbeck model can be written as the following differential equation:

dV=(λV+kI)dt+σdW

where *V* is the amount of accumulated evidence at time *t*, *dW* is independent white noise (Wiener), and *λ*, *k*, *σ* are free parameters. The process proceeds in steps of *dt* = 1ms. The parameters used to simulate the model [46] are:

*λ* = 2.3, *k* = 0.11*ms* ^−1^, *σ* = 0.6 and a non-decision time of 400ms.

## Supporting Information

S1 Text Supplementary Methods. (PDF)

S1 Fig Reaction-time histograms and quantile-utility plots for Tasks 1–4 respectively, pooling all of the data from each task. In the histograms, black circles indicate the subject means with standard error bars clustered by subject. Red dashed lines indicate the aDDM predictions. In the quantile-utility plots, circles indicate the aggregate subject data and lines indicate the DDM predictions (*θ* = 0.3) with lighter shading showing 95% confidence intervals from the simulations. Black, red, blue, green and gray colors indicate the 10%, 30%, 50%, 70% and 90% quantiles for reaction times.
(PDF)

S2 Fig Subjects’ mean and individually de-meaned reaction times in Tasks 1–3 as a function of the utility difference between the selfish option and the fair option. Black circles indicate the aggregate subject data with standard error bars clustered by subject. Red dashed lines indicate the aDDM predictions (*θ* = 0.3) with 95% confidence intervals. For Tasks 2 & 3, just as in Figs 3 and 4 of the main text, the blue dotted line indicates the inferior predictions of the alternative DDM with *θ* = 0.15 due to the presence of the co-dictator.
(PDF)

S3 Fig Log-likelihood estimates for the DDM choice curves as a function of the focus parameter θ, for Tasks 1–4 respectively. In the left column, each point represents the likelihood that the choice probabilities for every decision in the data were generated by an aDDM with a weight of *θ* on the other subject’s payoff. The right column is equivalent to the left column, except we varied both the weight between self and other payoff as well as the correction factor (otherwise 0.65) for non-eye-tracking data. The vertical dotted lines indicate the *θ* = 0.3 aDDM used in the main text and the gray shading indicates the interval *θ* = [0.25,0.35], which is the precision of the parameter θ estimated in the previous food choice studies.
(PDF)

S4 Fig Replications of the main text figures changing value of the self/other focus parameter *θ* to either 0.2 or 0.4. Panels A-D change from *θ* = 0.3 to 0.2. **A)** Task 1 (related to Fig 2 in main text), **B)** Task 2 (Fig 3), **C)** Task 3 (Fig 4), **D)** Task 4 (Fig 5). Panels E-H change from *θ* = 0.3 to 0.4. **E)** Task 1 (Fig 2), **F)** Task 2 (Fig 3), **G)** Task 3 (Fig 4), **H)** Task 4 (Fig 5). The model predictions using a focus parameter of 0.2 or 0.4 frequently mispredict the aggregate choice and RT values across the different tasks as can be seen by the lack of overlap between the 95% confidence intervals in blue and the empirical standard error bars in black. Misprediction with *θ* = 0.2 is particularly strong for the behaviors in Tasks 2–4 while mispredition with *θ* = 0.4 is particularly strong for behavior in Tasks 1–3.
(PDF)

S5 Fig Replications of the main text RT figures with the alternative Fehr-Schmidt and Bolton-Ockenfels models. **A)** Task 1 (related to Fig 2 in main text), **B)** Task 2 (related to Fig 3 in main text), **C)** Task 3 (related to Fig 4 in main text), **D)** Task 4 (related to Fig 5 in main text). Black circles indicate the aggregate subject data with bars representing s.e.m. Dashed lines indicate the alternative model predictions, while the solid red line indicates the predictions of the aDDM.
(PDF)

S6 Fig An example decision screen from Task 2. Note that the payoffs for Player A (the dictator) and Player B (the partner) are always the same in this task. The aDDM accurately predicts that the dictator will thus treat the game as if there were only two players, Player A and Player C (the receiver).
(PDF)

S7 Fig An example decision screen from Task 3. Note that in contrast to Task 2, there are only two columns of payoffs in this task.
(PDF)

S1 Table Goodness-of-fit statistics for the aDDM. ‘Error’ refers to the average difference between the model predictions and the actual data, as described in the main text. P-values refer to the more traditional goodness-of-fit measures, described in the methods. For reaction-time goodness of fits, the listed p-values are for the slope and intercept, respectively. When taking into account corrections for multiple-comparisons, none of the data reject the model.
(PDF)

S2 Table Value ranges and parameters used in the original food study and the social-preference Tasks 1–4 of the current study. (PDF)

S3 Table Payoffs for the dictator (self) and receiver (other) for the two options in Task 1. Points were converted to Swiss Francs at the end of the experiment.
(PDF)

S4 Table Payoffs for the dictators (self) and receiver (other) for the two options in Tasks 2 & 3. Points were converted to Swiss Francs at the end of the experiment.
(PDF)
