# Supplementary material for: A Common Mechanism Underlying Food Choice and Social Decisions
Source: PLoS Comput Biol. 2015 Oct 13;11(10):e1004371. doi: 10.1371/journal.pcbi.1004371 (PMC4604207; doi:10.1371/journal.pcbi.1004371)
Supplement: S1 Text — (PDF) [file pcbi.1004371.s001.pdf]

Supplementary Methods

Confidence interval calculations

95% confidence intervals for the model were calculated using a bootstrap method with 1000 simulated datasets equal in size to the real data.

Goodness-of-fit calculations

For the choice figures we used chi-squared tests to compare the predicted and observed proportions of choices for each bin (as indicated in the figures). For the reaction-time figures we used a goodness-of-fit statistic from our previous papers. For each reaction-time figure we calculate the residual curve by subtracting the DDM prediction from the data. If the simulations fit the data well, then the residuals should be a flat line at 0. We test this prediction using an ordinary least squares (OLS) regression model and so for the goodness of fits we report the *p*-values for the coefficients of the regression.

Indifference-point plots

To examine the relationship between choice difficulty and reaction time in Task 1 and Task 2 we converted the pair of objective payoffs for each choice option into a single subjective value (utility) using the payoff weightings from the aDDM. Formally, for each social allocation (x_i = own payoff, x_j = others' payoffs) we calculated the utility as:

$$U_i(X) = x_i + 0.3x_j$$

Choices for which $U(X) - U(Y) = 0$ represent the most difficult choices, i.e. choices where subjects are on average indifferent between choosing options X and Y. As the magnitude of this utility difference increases, the decisions become easier. The aDDM predicts that reaction times will peak at indifference and then decrease as decisions become easier. In Figure S2 we verify this prediction by plotting mean reaction times against the utility-difference measure.

Quantile utility plots

Following the standard convention, for each task we created quantile-utility plots, showing the predicted and actual reaction times for the 0.1, 0.3, 0.5 (median), 0.7, and 0.9 quantiles as a function of utility (defined immediately above; see Fig. S1).
