# Supplementary material for: A Common Mechanism Underlying Food Choice and Social Decisions
Source: PLoS Comput Biol. 2015 Oct 13;11(10):e1004371. doi: 10.1371/journal.pcbi.1004371 (PMC4604207; doi:10.1371/journal.pcbi.1004371)
Supplement: S1 Fig — In the histograms, black circles indicate the subject means with standard error bars clustered by subject. Red dashed lines indicate the aDDM predictions. In the quantile-utility plots, circles indicate the aggregate subject data and lines indicate the DDM predictions (θ = 0.3) with lighter shading showing 95% confidence intervals from the simulations. Black, red, blue, green and gray colors indicate the 10%, 30%, 50%, 70% and 90% quantiles for reaction times. (PDF) [file pcbi.1004371.s002.pdf]

Task 1

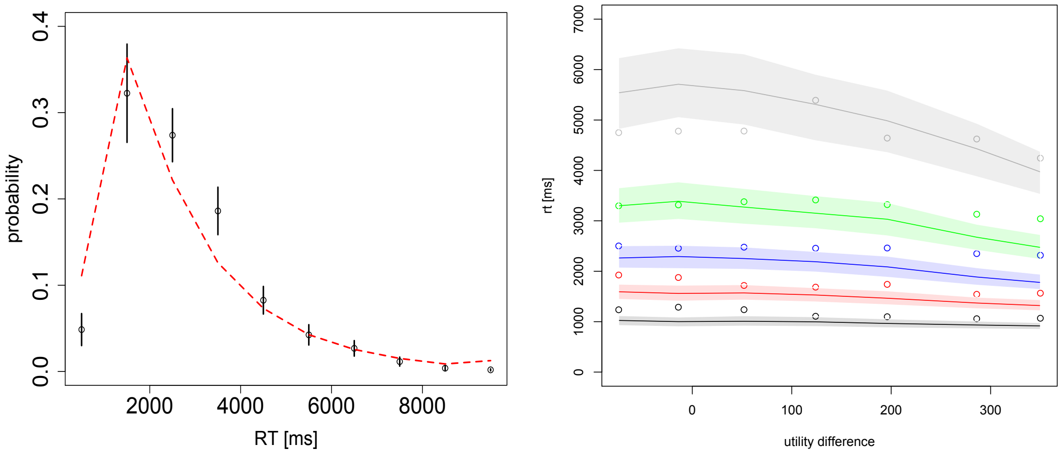

Task 2

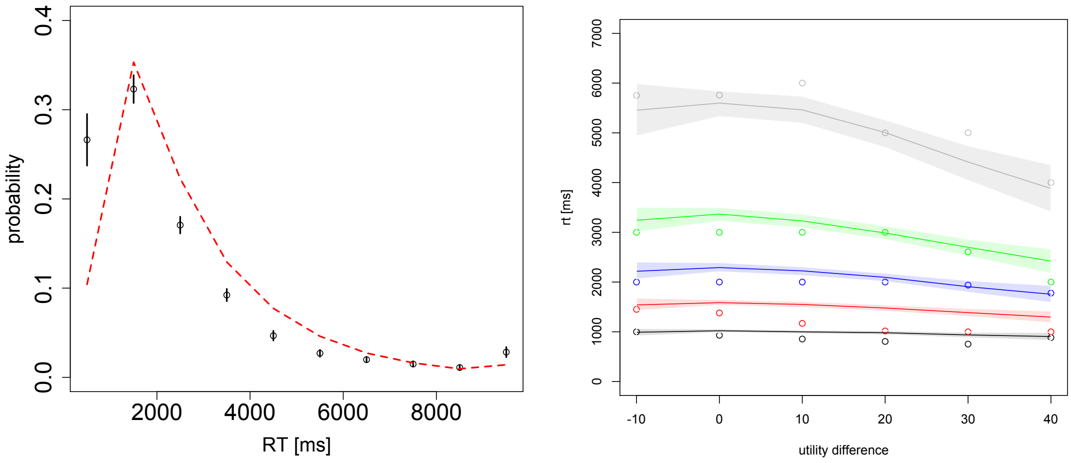

Task 3

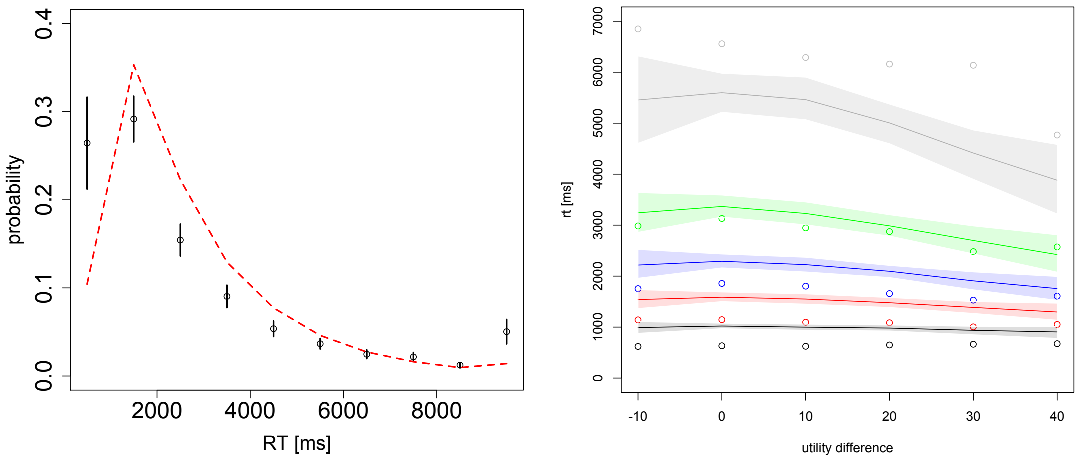

Task 4

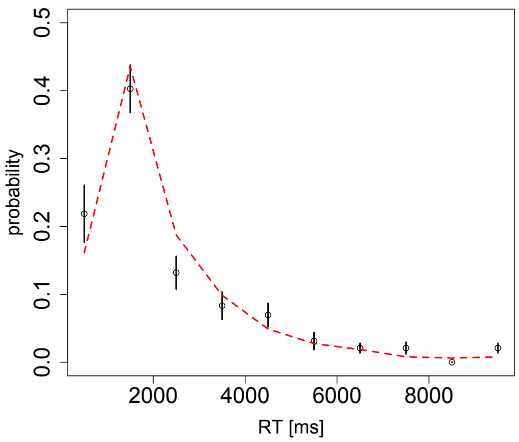

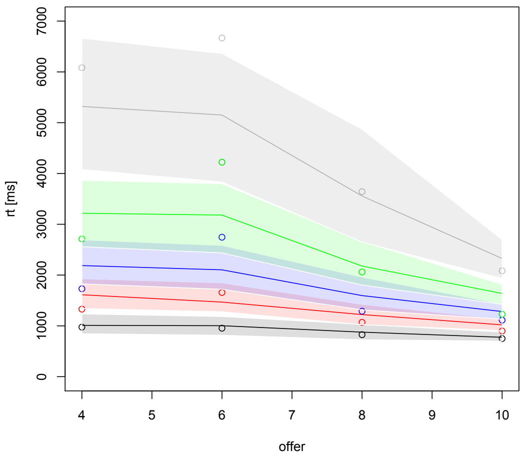

Figure S1: Reaction-time histograms and quantile-utility plots for Tasks 1-4 respectively, pooling all of the data from each task. In the histograms, black circles indicate the subject means with standard error bars clustered by subject. Red dashed lines indicate the aDDM predictions. In the quantile-utility plots, circles indicate the aggregate subject data and lines indicate the DDM predictions ($\theta = 0.3$) with lighter shading showing 95% confidence intervals from the simulations. Black, red, blue, green and gray colors indicate the 10%, 30%, 50%, 70% and 90% quantiles for reaction times.
