# Supplementary material for: A Common Mechanism Underlying Food Choice and Social Decisions
Source: PLoS Comput Biol. 2015 Oct 13;11(10):e1004371. doi: 10.1371/journal.pcbi.1004371 (PMC4604207; doi:10.1371/journal.pcbi.1004371)
Supplement: S2 Fig — Black circles indicate the aggregate subject data with standard error bars clustered by subject. Red dashed lines indicate the aDDM predictions (θ = 0.3) with 95% confidence intervals. For Tasks 2 & 3, just as in Figs 3 and 4 of the main text, the blue dotted line indicates the inferior predictions of the alternative DDM with θ = 0.15 due to the presence of the co-dictator. (PDF) [file pcbi.1004371.s003.pdf]

Task 1

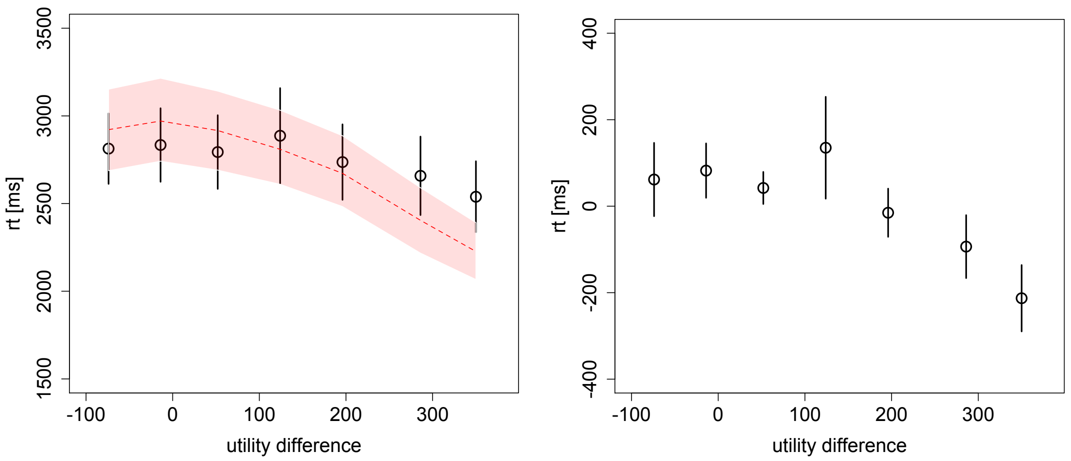

Task 2

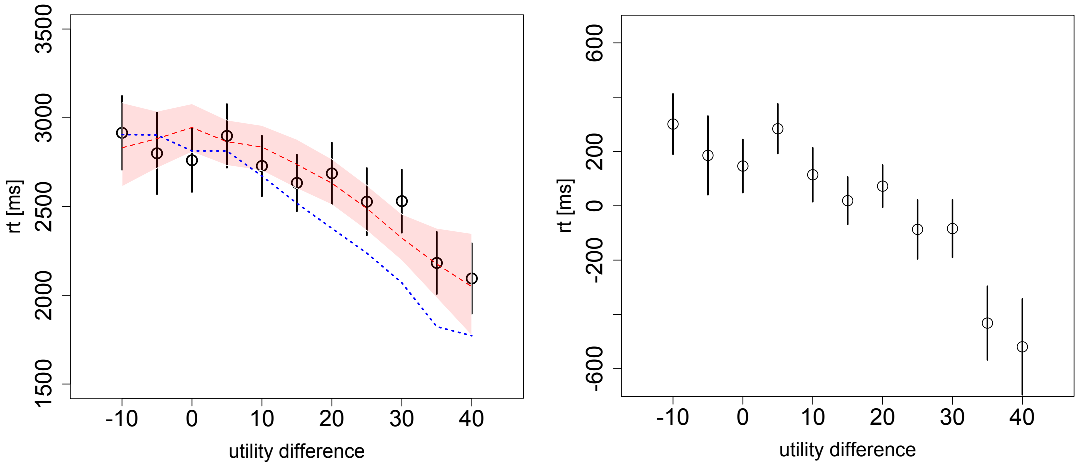

Task 3

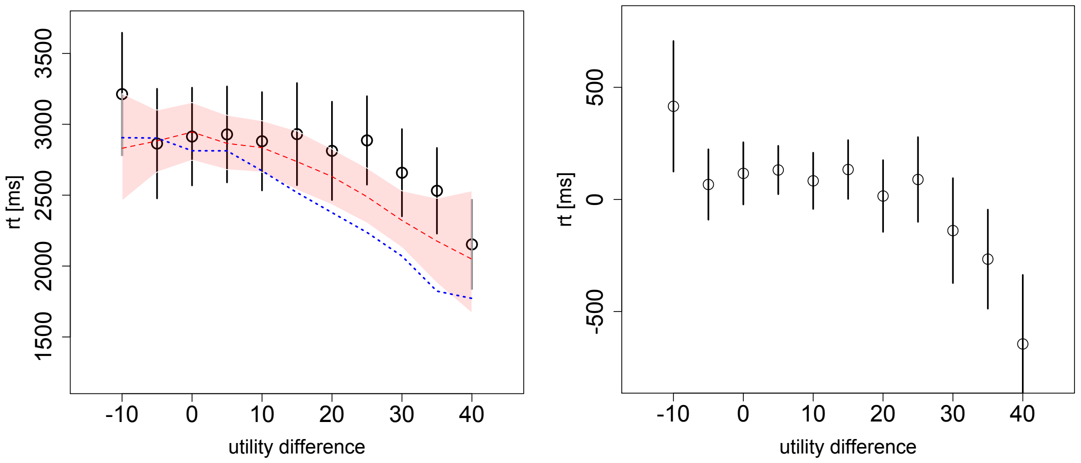

Figure S2: Subjects' mean and individually de-meaned reaction times in Tasks 1-3 as a function of the utility difference between the selfish option and the fair option. Black circles indicate the aggregate subject data with standard error bars clustered by subject. Red dashed lines indicate the aDDM predictions ($\theta = 0.3$) with 95% confidence intervals. For Tasks 2 & 3, just as in Figures 3 & 4 of the main text, the blue dotted line indicates the inferior predictions of the alternative DDM with $\theta = 0.15$ due to the presence of the co-dictator.
