# Supplementary material for: A Common Mechanism Underlying Food Choice and Social Decisions
Source: PLoS Comput Biol. 2015 Oct 13;11(10):e1004371. doi: 10.1371/journal.pcbi.1004371 (PMC4604207; doi:10.1371/journal.pcbi.1004371)
Supplement: S3 Fig — In the left column, each point represents the likelihood that the choice probabilities for every decision in the data were generated by an aDDM with a weight of θ on the other subject’s payoff. The right column is equivalent to the left column, except we varied both the weight between self and other payoff as well as the correction factor (otherwise 0.65) for non-eye-tracking data. The vertical dotted lines indicate the θ = 0.3 aDDM used in the main text and the gray shading indicates the interval θ = [0.25,0.35], which is the precision of the parameter θ estimated in the previous food choice studies. (PDF) [file pcbi.1004371.s004.pdf]

Task 1

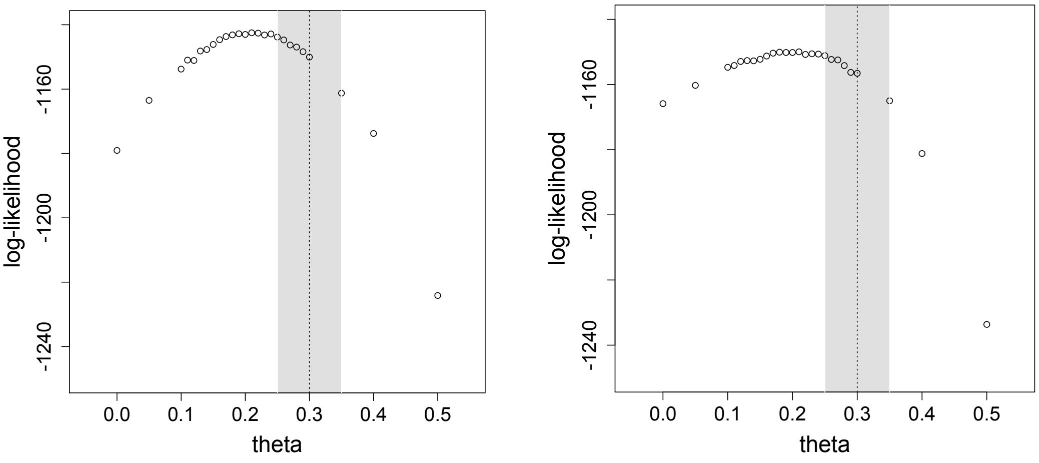

Task 2

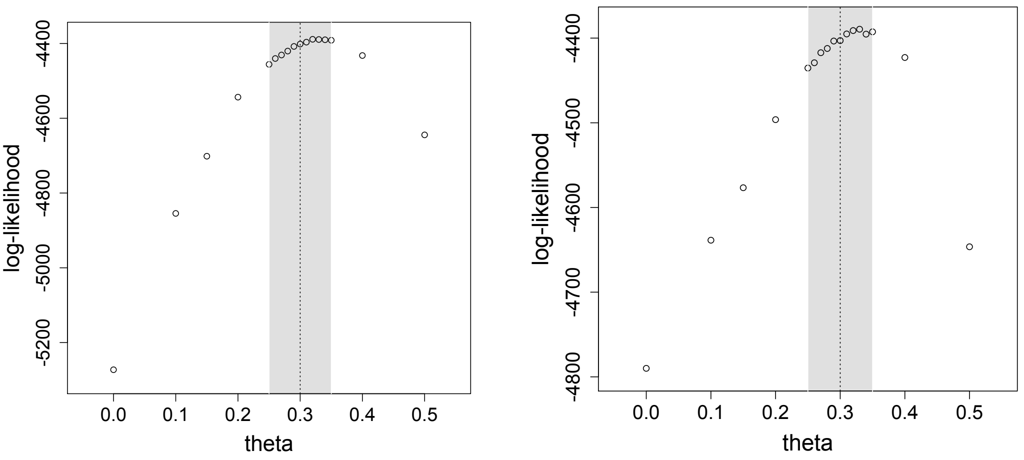

Task 3

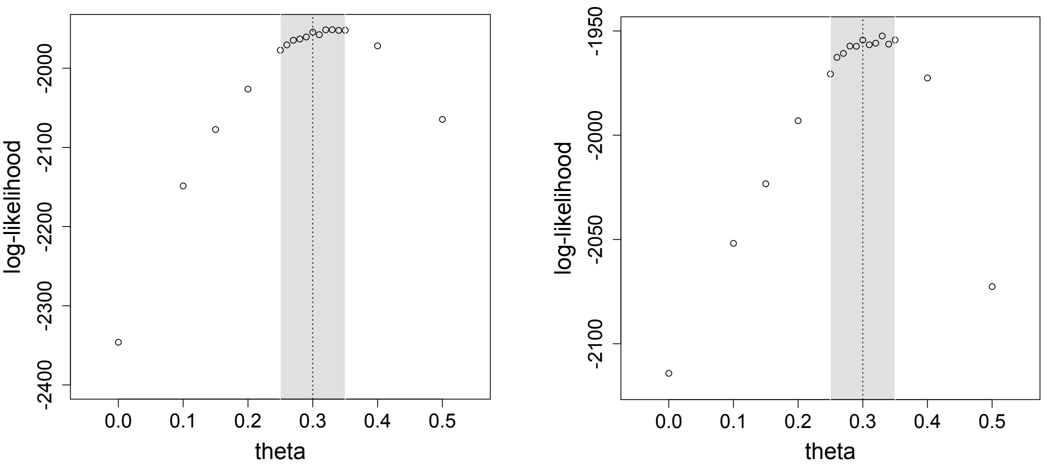

Task 4

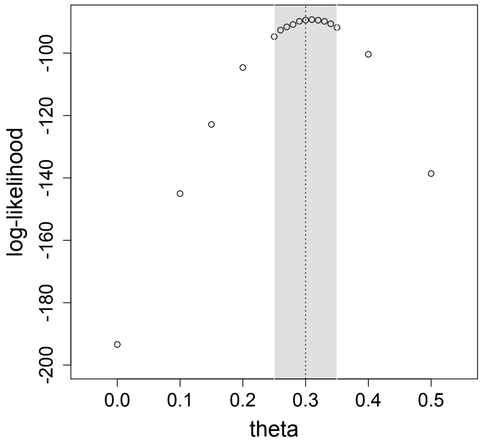

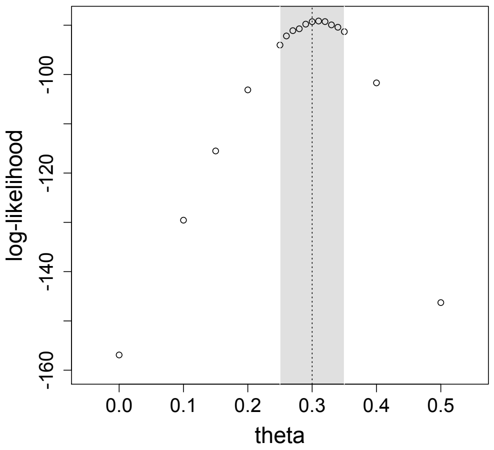

Figure S3: Log-likelihood estimates for the DDM choice curves as a function of the focus parameter θ, for Tasks 1-4 respectively. In the left column, each point represents the likelihood that the choice probabilities for every decision in the data were generated by an aDDM with a weight of θ on the other subject's payoff. The right column is equivalent to the left column, except we varied both the weight between self and other payoff as well as the correction factor (otherwise 0.65) for non-eye-tracking data. The vertical dotted lines indicate the θ=0.3 aDDM used in the main text and the gray shading indicates the interval θ=[0.25,0.35], which is the precision of the parameter θ estimated in the previous food choice studies.
