# Supplementary material for: A Common Mechanism Underlying Food Choice and Social Decisions
Source: PLoS Comput Biol. 2015 Oct 13;11(10):e1004371. doi: 10.1371/journal.pcbi.1004371 (PMC4604207; doi:10.1371/journal.pcbi.1004371)
Supplement: S4 Fig — A) Task 1 (related to Fig 2 in main text), B) Task 2 (Fig 3), C) Task 3 (Fig 4), D) Task 4 (Fig 5). Panels E-H change from θ = 0.3 to 0.4. E) Task 1 (Fig 2), F) Task 2 (Fig 3), G) Task 3 (Fig 4), H) Task 4 (Fig 5). The model predictions using a focus parameter of 0.2 or 0.4 frequently mispredict the aggregate choice and RT values across the different tasks as can be seen by the lack of overlap between the 95% confidence intervals in blue and the empirical standard error bars in black. Misprediction with θ = 0.2 is particularly strong for the behaviors in Tasks 2–4 while mispredition with θ = 0.4 is particularly strong for behavior in Tasks 1–3. (PDF) [file pcbi.1004371.s005.pdf]

A

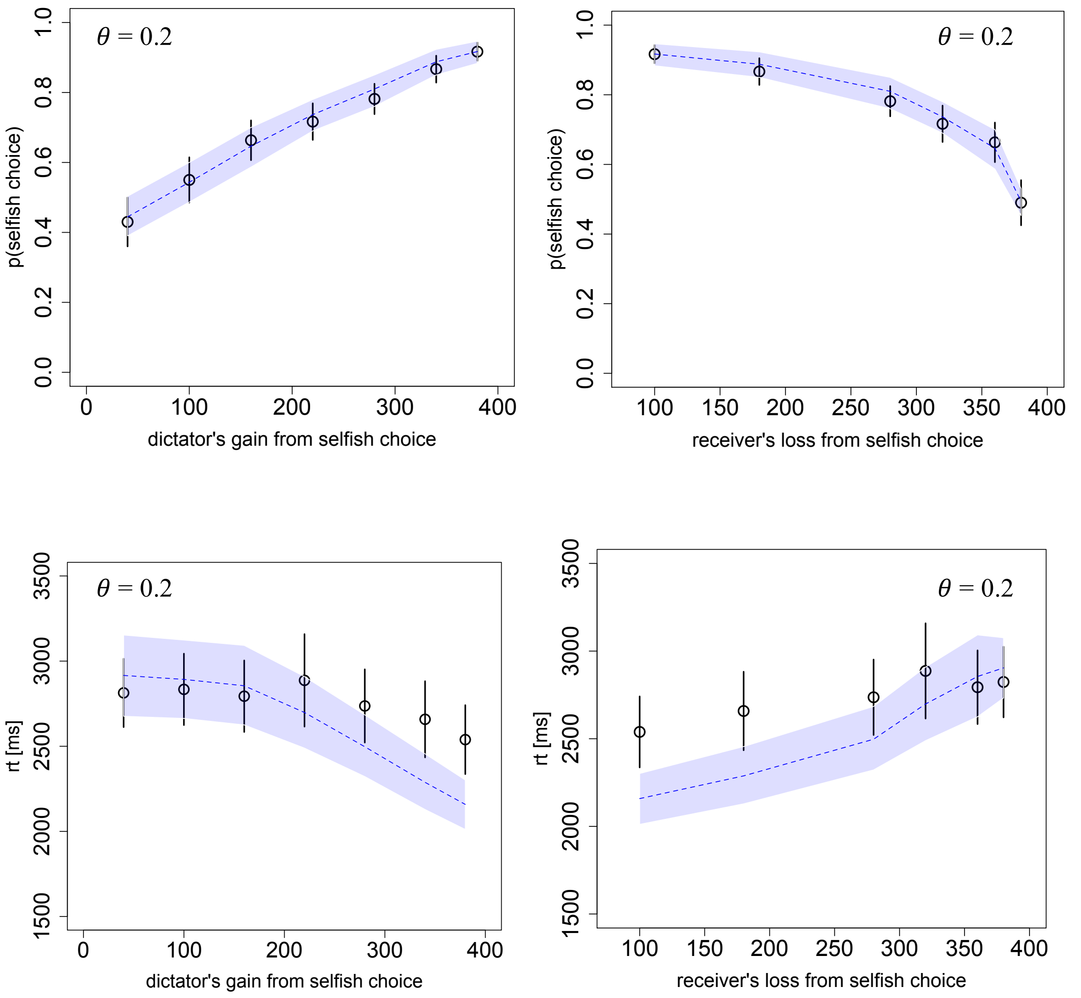
θ = 0.2
p(selfish choice)
0.0
0.2
0.4
0.6
0.8
1.0
0
100
200
300
400
dictator's gain from selfish choice
θ = 0.2
p(selfish choice)
100
150
200
250
300
350
400
receiver's loss from selfish choice
θ = 0.2
rt [ms]
1500
2000
2500
3000
3500
dictator's gain from selfish choice
θ = 0.2
rt [ms]
receiver's loss from selfish choice

B

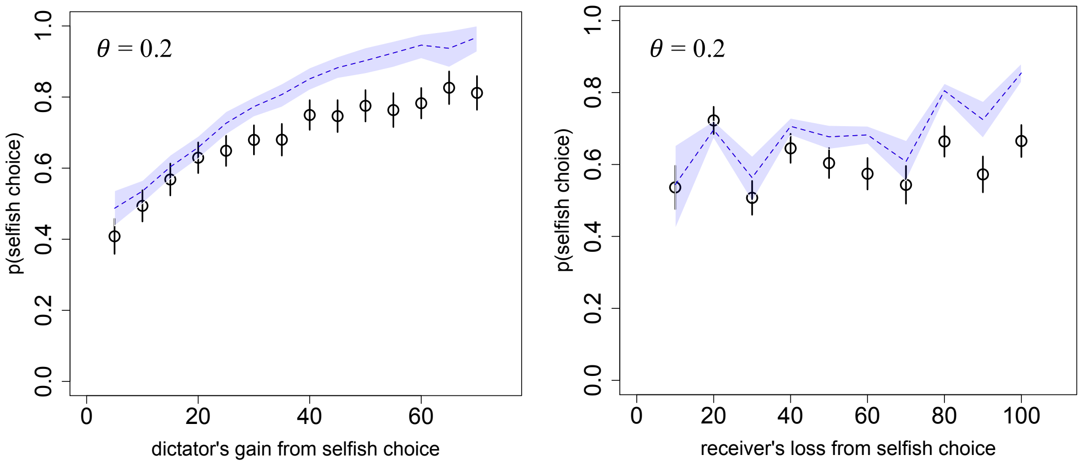
θ = 0.2
p(selfish choice)
0.0
0.2
0.4
0.6
0.8
1.0
0
20
40
60
dictator's gain from selfish choice
θ = 0.2
p(selfish choice)
0
20
40
60
80
100
receiver's loss from selfish choice

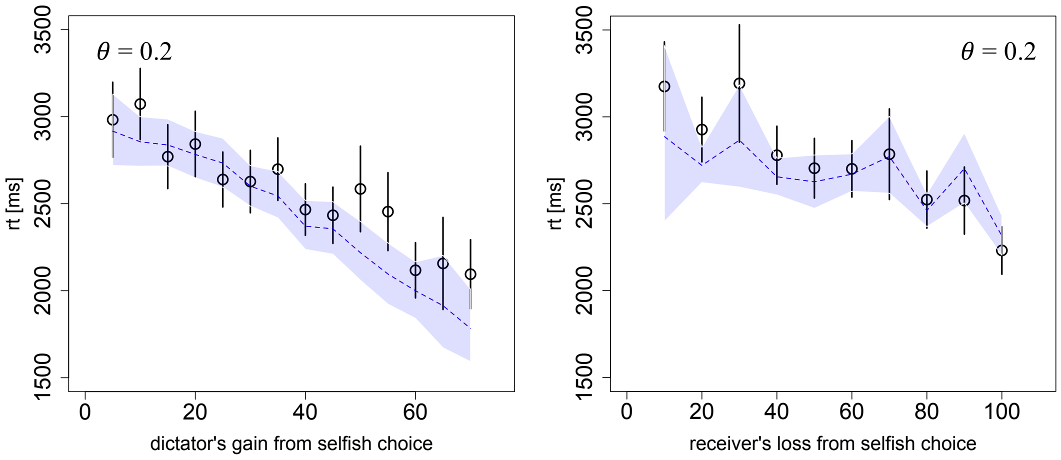
θ = 0.2
rt [ms]
dictator's gain from selfish choice
θ = 0.2
rt [ms]
receiver's loss from selfish choice

C

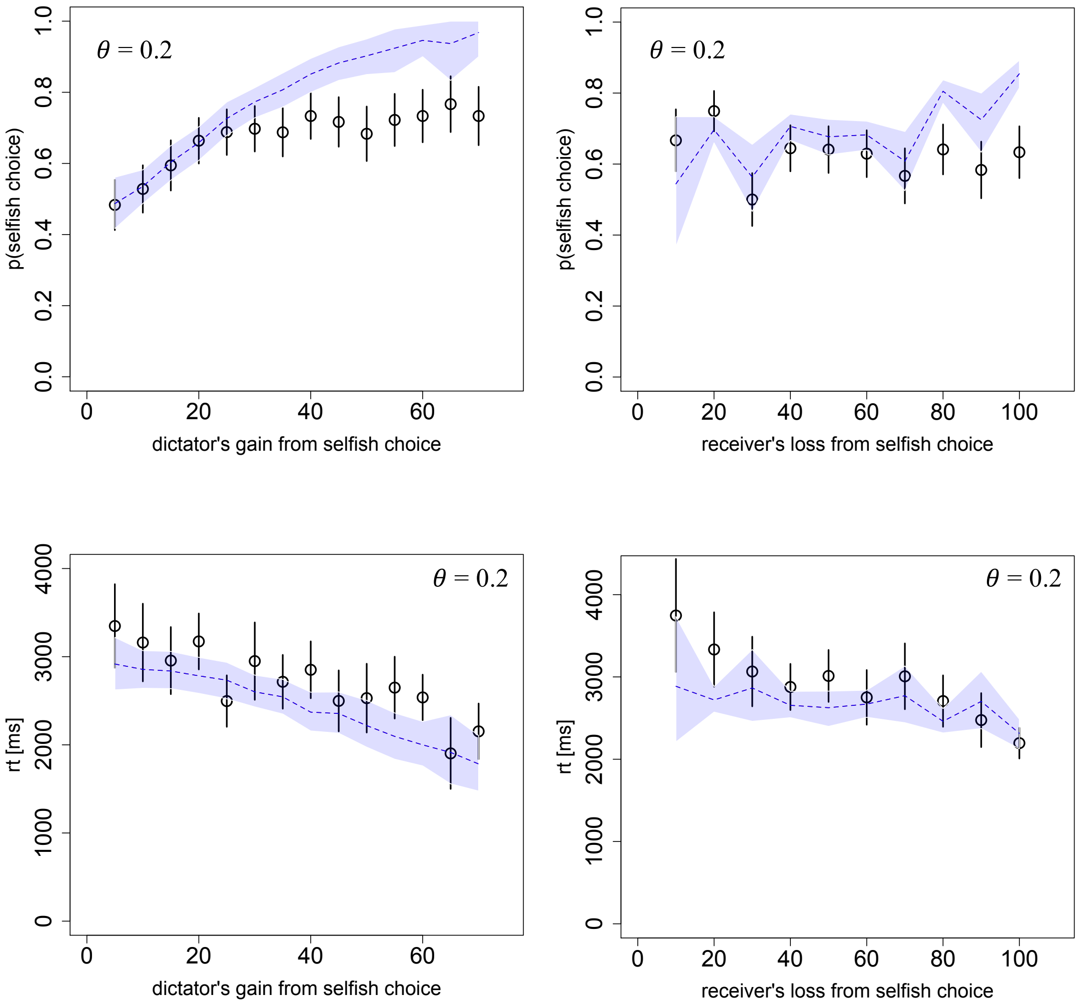
θ = 0.2
p(selfish choice)
dictator's gain from selfish choice
θ = 0.2
p(selfish choice)
receiver's loss from selfish choice
θ = 0.2
rt [ms]
dictator's gain from selfish choice
θ = 0.2
rt [ms]
receiver's loss from selfish choice

D

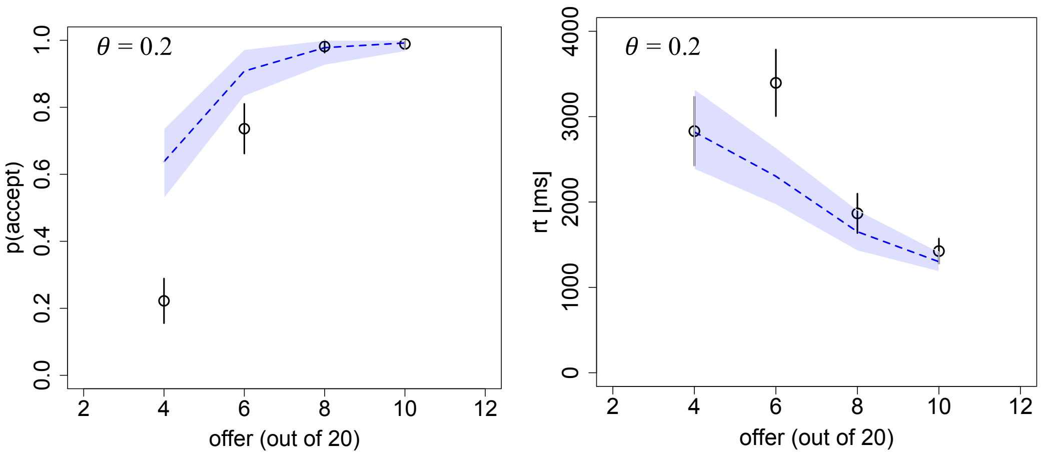

θ = 0.2
p(accept)
offer (out of 20)
θ = 0.2
rt [ms]
offer (out of 20)

E

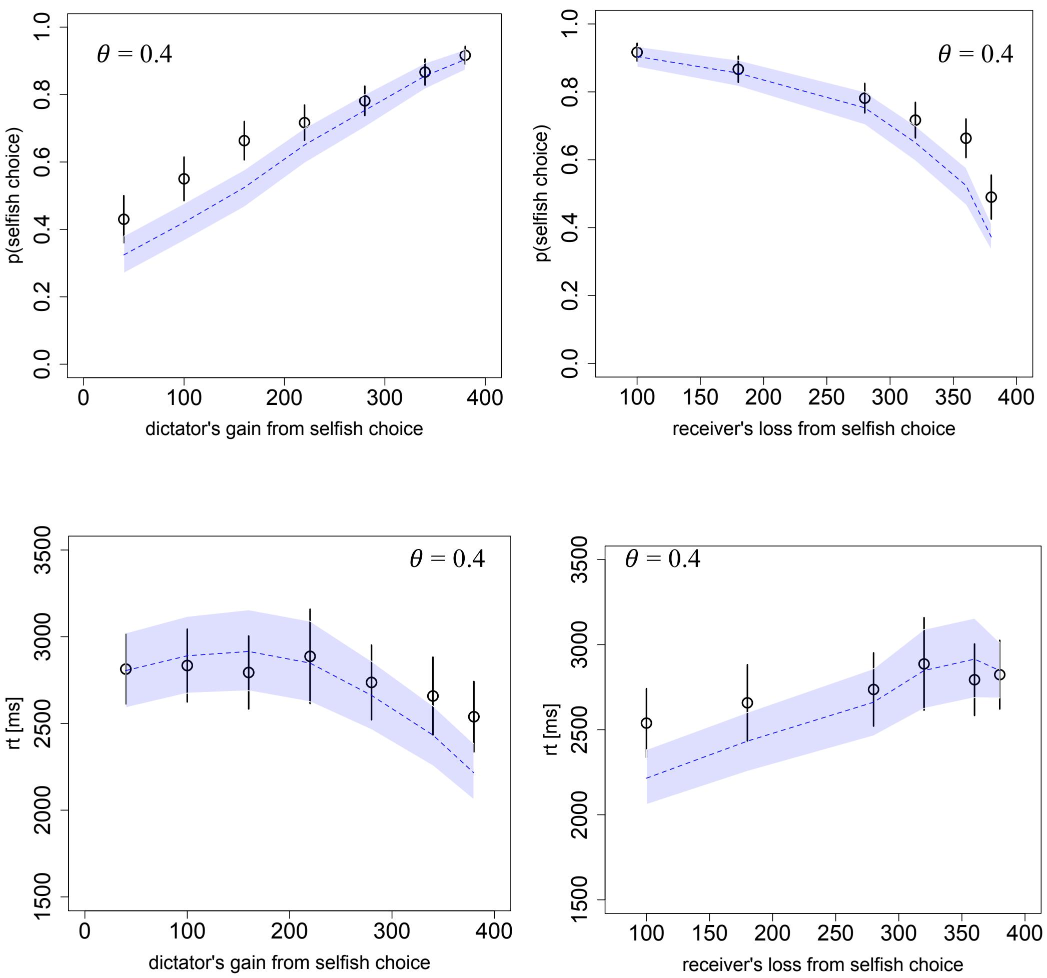

θ = 0.4
p(selfish choice)
dictator's gain from selfish choice
θ = 0.4
p(selfish choice)
receiver's loss from selfish choice
θ = 0.4
rt [ms]
dictator's gain from selfish choice
θ = 0.4
rt [ms]
receiver's loss from selfish choice

F

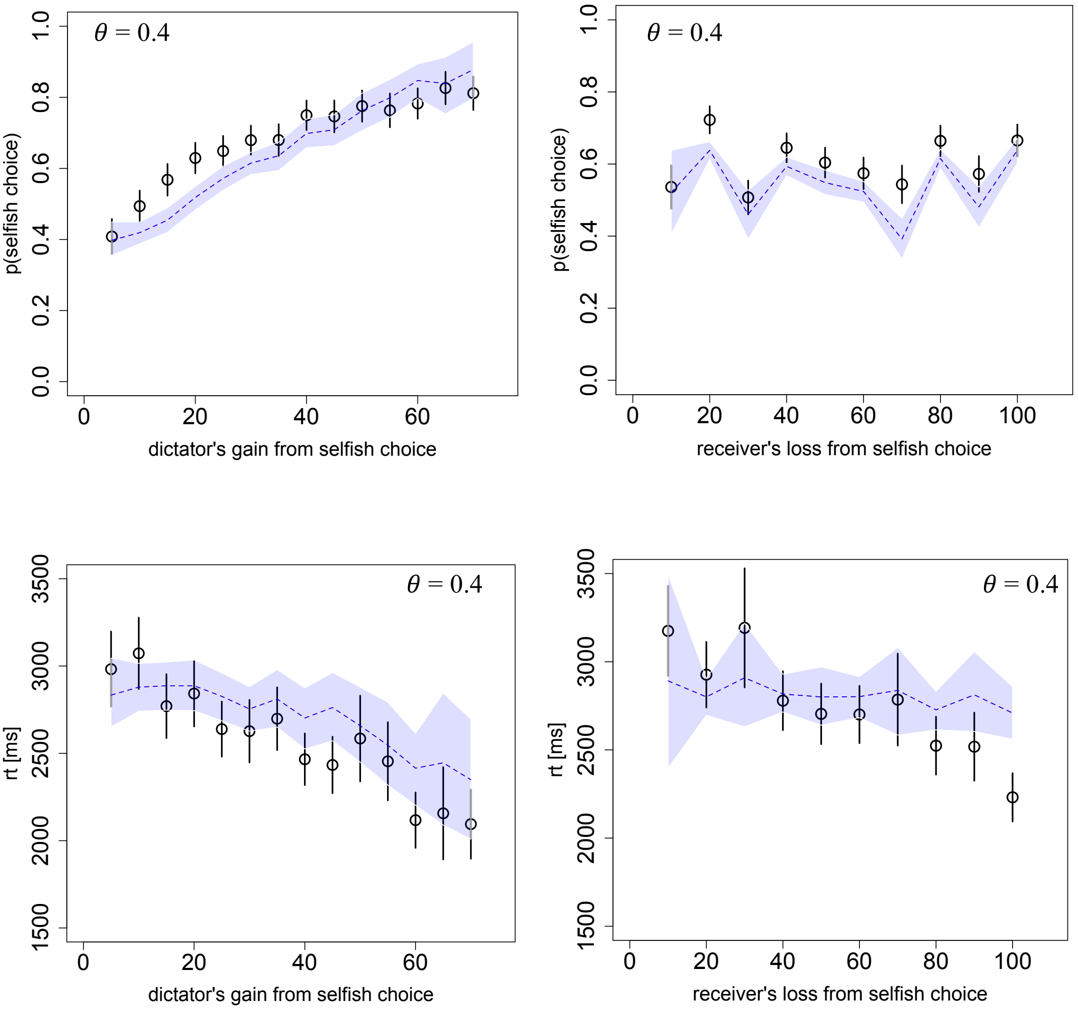
$\theta = 0.4$
p(selfish choice)
dictator's gain from selfish choice
$\theta = 0.4$
p(selfish choice)
receiver's loss from selfish choice
$\theta = 0.4$
rt [ms]
dictator's gain from selfish choice
$\theta = 0.4$
rt [ms]
receiver's loss from selfish choice

G

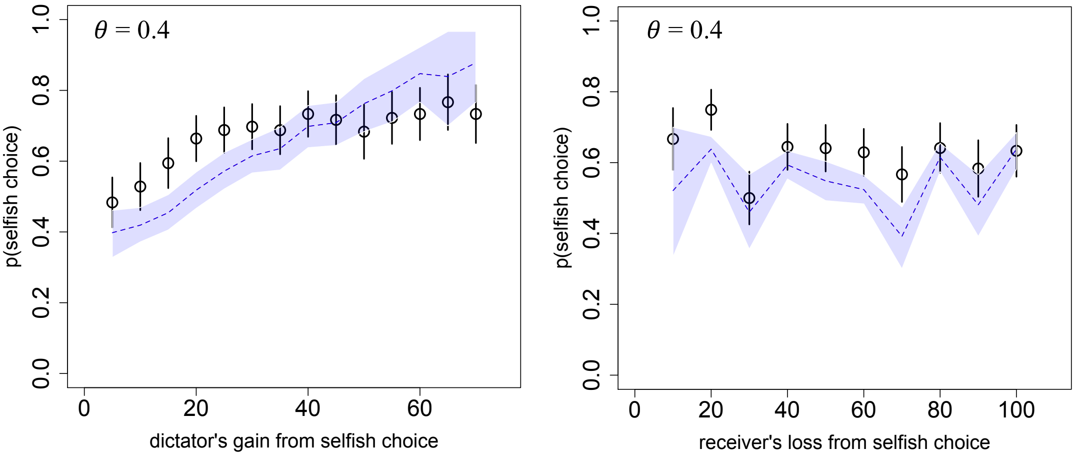
$\theta = 0.4$
p(selfish choice)
dictator's gain from selfish choice
$\theta = 0.4$
p(selfish choice)
receiver's loss from selfish choice

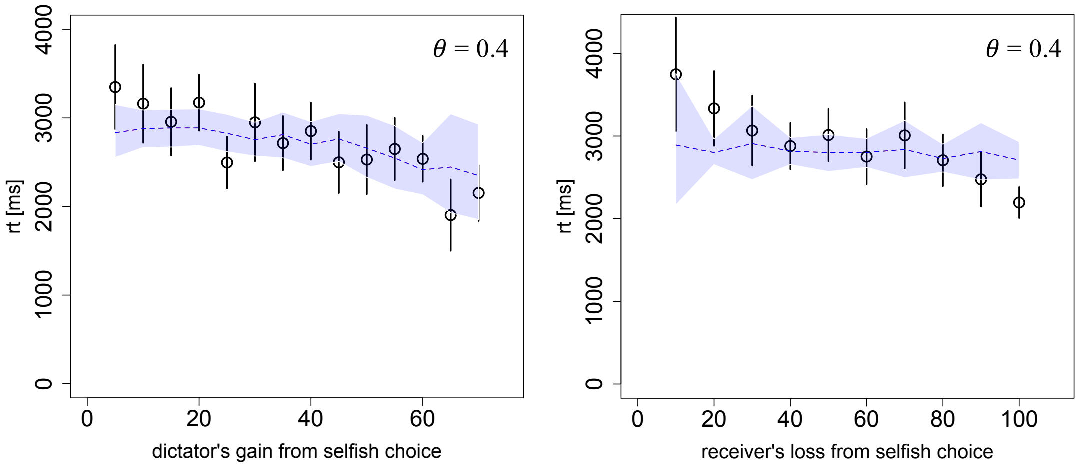

H

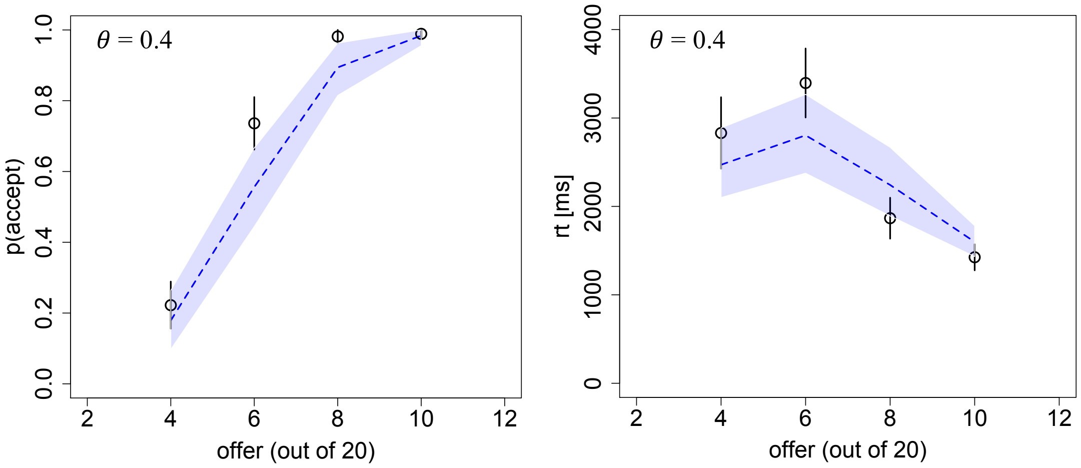

Figure S4: Replications of the main text figures changing value of the self/other focus parameter θ to either 0.2 or 0.4. Panels A-D change from $\theta = 0.3$ to 0.2. **A)** Task 1 (related to Fig. 2 in main text), **B)** Task 2 (Fig. 3), **C)** Task 3 (Fig. 4), **D)** Task 4 (Fig. 5). Panels E-H change from $\theta = 0.3$ to 0.4. **E)** Task 1 (Fig. 2), **F)** Task 2 (Fig. 3), **G)** Task 3 (Fig. 4), **H)** Task 4 (Fig. 5). The model predictions using a focus parameter of 0.2 or 0.4 frequently mispredict the aggregate choice and RT values across the different tasks as can be seen by the lack of overlap between the 95% confidence intervals in blue and the empirical standard error bars in black. Misprediction with $\theta = 0.2$ is particularly strong for the behaviors in Tasks 2-4 while mispredition with $\theta = 0.4$ is particularly strong for behavior in Tasks 1-3.
