# Supplementary material for: A Common Mechanism Underlying Food Choice and Social Decisions
Source: PLoS Comput Biol. 2015 Oct 13;11(10):e1004371. doi: 10.1371/journal.pcbi.1004371 (PMC4604207; doi:10.1371/journal.pcbi.1004371)
Supplement: S5 Fig — A) Task 1 (related to Fig 2 in main text), B) Task 2 (related to Fig 3 in main text), C) Task 3 (related to Fig 4 in main text), D) Task 4 (related to Fig 5 in main text). Black circles indicate the aggregate subject data with bars representing s.e.m. Dashed lines indicate the alternative model predictions, while the solid red line indicates the predictions of the aDDM. (PDF) [file pcbi.1004371.s006.pdf]

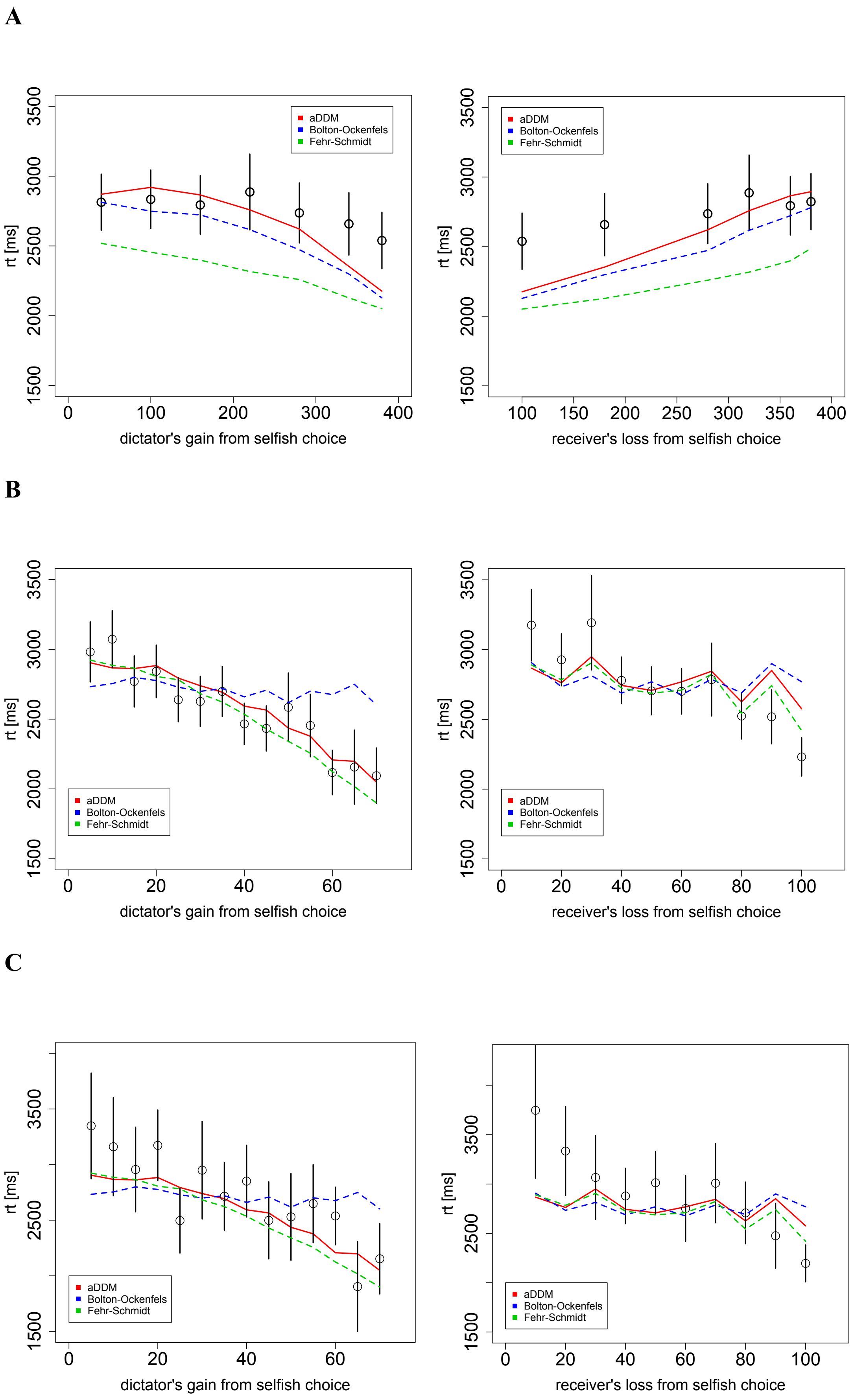
A
B
C
aDDM
Bolton-Ockenfels
Fehr-Schmidt
rt [ms]
1500
2000
2500
3000
3500
0
100
200
300
400
150
250
350
20
40
60
80
dictator's gain from selfish choice
receiver's loss from selfish choice

D

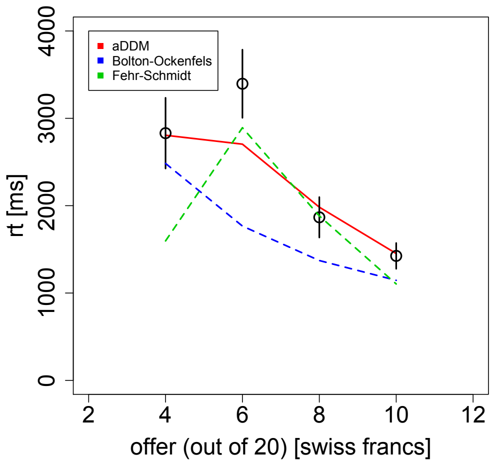

Figure S5: Replications of the main text RT figures with the alternative Fehr-Schmidt and Bolton-Ockenfels models **A)** Task 1 (related to Fig. 2 in main text), **B)** Task 2 (related to Fig. 3 in main text), **C)** Task 3 (related to Fig. 4 in main text), **D)** Task 4 (related to Fig. 5 in main text). Black circles indicate the aggregate subject data with bars representing s.e.m. Dashed lines indicate the alternative model predictions, while the solid red line indicates the predictions of the aDDM.
