# Supplementary material for: A Common Mechanism Underlying Food Choice and Social Decisions
Source: PLoS Comput Biol. 2015 Oct 13;11(10):e1004371. doi: 10.1371/journal.pcbi.1004371 (PMC4604207; doi:10.1371/journal.pcbi.1004371)
Supplement: S6 Fig — Note that the payoffs for Player A (the dictator) and Player B (the partner) are always the same in this task. The aDDM accurately predicts that the dictator will thus treat the game as if there were only two players, Player A and Player C (the receiver). (PDF) [file pcbi.1004371.s007.pdf]

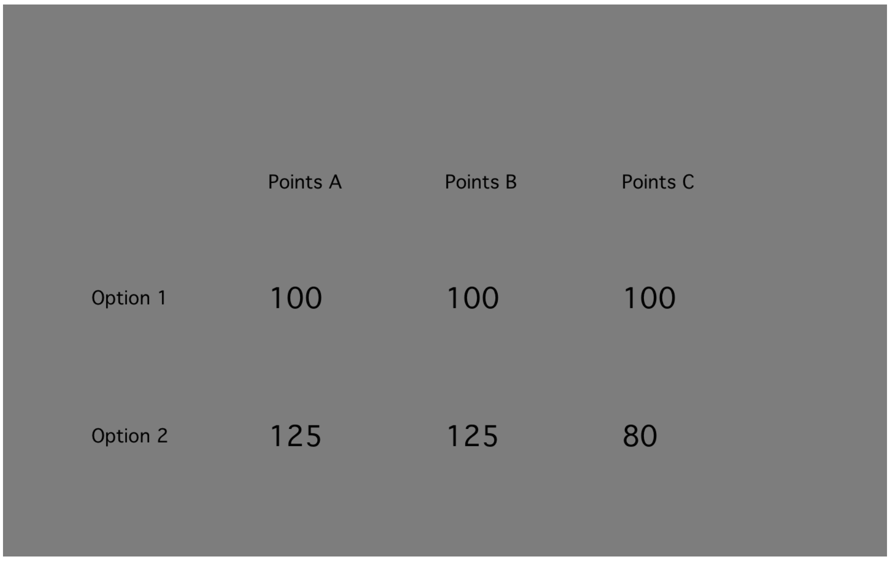

Figure S6: An example decision screen from Task 2. Note that the payoffs for Player A (the dictator) and Player B (the partner) are always the same in this task. The aDDM accurately predicts that the dictator will thus treat the game as if there were only two players, Player A and Player C (the receiver).
