# Supplementary material for: A Common Mechanism Underlying Food Choice and Social Decisions
Source: PLoS Comput Biol. 2015 Oct 13;11(10):e1004371. doi: 10.1371/journal.pcbi.1004371 (PMC4604207; doi:10.1371/journal.pcbi.1004371)
Supplement: S7 Fig — Note that in contrast to Task 2, there are only two columns of payoffs in this task. (PDF) [file pcbi.1004371.s008.pdf]

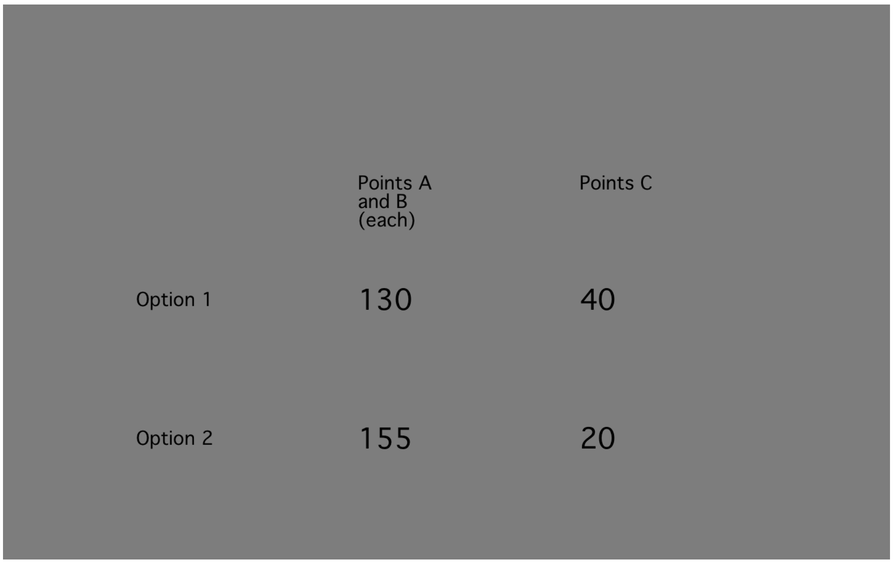

Figure S7: An example decision screen from Task 3. Note that in contrast to Task 2, there are only two columns of payoffs in this task.
