# Supplementary material for: A Common Mechanism Underlying Food Choice and Social Decisions
Source: PLoS Comput Biol. 2015 Oct 13;11(10):e1004371. doi: 10.1371/journal.pcbi.1004371 (PMC4604207; doi:10.1371/journal.pcbi.1004371)
Supplement: S1 Table — ‘Error’ refers to the average difference between the model predictions and the actual data, as described in the main text. P-values refer to the more traditional goodness-of-fit measures, described in the methods. For reaction-time goodness of fits, the listed p-values are for the slope and intercept, respectively. When taking into account corrections for multiple-comparisons, none of the data reject the model. (PDF) [file pcbi.1004371.s009.pdf]

Goodness of fit	aDDM fit
Fig. 2a error	3.07%
Fig. 2a χ^2 (p-value)	3.93 (0.69)
Fig. 2b error	2.67%
Fig. 2b χ^2 (p-value)	3.93 (0.56)
Fig. 2c error	5.57%
Fig. 2c p-values	0.06, 0.4
Fig. 3a error	5.61%
Fig. 3a χ^2 (p-value)	16.35 (0.23)
Fig. 3b error	3.42%
Fig. 3b χ^2 (p-value)	18.18 (0.03)
Fig. 3c error	3.14%
Fig. 3c p-values	0.87, 1
Fig. 3d error	5.20%
Fig. 3d p-values	0.03, 0.03
Fig. 4a error	9.06%
Fig. 4a χ^2 (p-value)	22.41 (0.05)
Fig. 4b error	6.55%
Fig. 4b χ^2 (p-value)	17.87 (0.04)
Fig. 4c error	5.61%
Fig. 4c p-values	0.49, 0.22
Fig. 4d error	8.07%
Fig. 4d p-values	0.10, 0.36
Fig. 4a error	5.65%
Fig. 4a χ^2 (p-value)	4.42 (0.22)
Fig. 4b error	6.32%
Fig. 4b p-values	0.5, 0.31

Table S1: Goodness-of-fit statistics for the aDDM. 'Error' refers to the average difference between the model predictions and the actual data, as described in the main text. P-values refer to the more traditional goodness-of-fit measures, described in the methods. For reaction-time goodness of fits, the listed p-values are for the slope and intercept, respectively. When taking into account corrections for multiple-comparisons, none of the data reject the model.
