# Supplementary material for: A Common Mechanism Underlying Food Choice and Social Decisions
Source: PLoS Comput Biol. 2015 Oct 13;11(10):e1004371. doi: 10.1371/journal.pcbi.1004371 (PMC4604207; doi:10.1371/journal.pcbi.1004371)
Supplement: S2 Table — (PDF) [file pcbi.1004371.s010.pdf]

	Actual self payoff range	Payoffs' order of magnitude $\bar{v}$	d	$d \cdot \bar{v}$	σ	θ
Food task	0 – 10	10	0.0002	**0.002**	**0.02**	**0.3**
Task 1	240 – 1170	1000	0.000002	**0.002**	**0.02**	**0.3**
Task 2	100 – 170	100	0.00002	**0.002**	**0.02**	**0.3**
Task 3	100 – 170	100	0.00002	**0.002**	**0.02**	**0.3**
Task 4	0 –10	10	0.0002	**0.002**	**0.02**	**-0.3**

Table S2: Value ranges and parameters used in the original food study and the social-preference Tasks 1-4 of the current study.
