# Supplementary material for: A Common Mechanism Underlying Food Choice and Social Decisions
Source: PLoS Comput Biol. 2015 Oct 13;11(10):e1004371. doi: 10.1371/journal.pcbi.1004371 (PMC4604207; doi:10.1371/journal.pcbi.1004371)
Supplement: S3 Table — Points were converted to Swiss Francs at the end of the experiment. (PDF) [file pcbi.1004371.s011.pdf]

Self Unfair	Self Fair	Other Unfair	Other Fair
1000	960	30	410
1000	960	270	650
800	760	-410	-30
560	520	-650	-270
480	440	550	930
760	720	30	410
760	720	270	650
240	200	790	1170
240	200	550	930
480	440	790	1170
790	690	30	410
790	690	270	650
590	490	-650	-270
510	410	550	930
1030	930	30	410
270	170	550	930
510	410	790	1170
830	730	-410	-30
1030	930	270	650
270	170	790	1170
540	380	560	920
860	700	-400	-40
620	460	-640	-280
820	660	40	400
820	660	280	640
1060	900	280	640
540	380	800	1160
300	140	560	920
1060	900	40	400
300	140	800	1160
570	350	820	1140
330	110	820	1140
850	630	300	620
1090	870	300	620
1090	870	60	380
330	110	580	900
570	350	580	900
650	430	-620	-300
850	630	60	380
890	670	-380	-60
680	400	-600	-320
880	600	320	600
1120	840	320	600
360	80	600	880
600	320	840	1120

880	600	80	360
360	80	840	1120
600	320	600	880
1120	840	80	360
920	640	-360	-80
390	50	650	830
1150	810	370	550
910	570	370	550
950	610	-310	-130
1150	810	130	310
390	50	890	1070
630	290	890	1070
710	370	-550	-370
910	570	130	310
630	290	650	830
650	270	690	790
650	270	930	1030
970	590	-270	-170
930	550	170	270
1170	790	170	270
1170	790	410	510
410	30	690	790
730	350	-510	-410
930	550	410	510
410	30	930	1030

Table S3: Payoffs for the dictator (self) and receiver (other) for the two options in Task 1. Points were converted to Swiss Francs at the end of the experiment.
