# Supplementary material for: A Common Mechanism Underlying Food Choice and Social Decisions
Source: PLoS Comput Biol. 2015 Oct 13;11(10):e1004371. doi: 10.1371/journal.pcbi.1004371 (PMC4604207; doi:10.1371/journal.pcbi.1004371)
Supplement: S4 Table — Points were converted to Swiss Francs at the end of the experiment. (PDF) [file pcbi.1004371.s012.pdf]

Self Fair	Other Fair	Self Unfair	Other Unfair
100	100	155	20
110	80	125	60
100	100	145	20
100	100	120	40
110	80	125	10
100	100	135	0
110	80	155	0
100	100	140	40
130	40	145	0
100	100	145	10
110	80	125	40
120	60	130	40
120	60	130	0
100	100	130	60
110	80	140	60
100	100	135	30
110	80	120	40
130	40	155	0
130	40	155	20
100	100	130	10
130	40	140	20
110	80	150	30
100	100	170	0
130	40	170	0
100	100	160	0
130	40	135	0
110	80	140	20
110	80	135	20
130	40	150	20
110	80	155	30
100	100	115	70
110	80	130	60
110	80	125	20
140	20	150	0
130	40	160	0
100	100	145	0
110	80	145	30
100	100	110	80
100	100	130	0
100	100	125	40
130	40	160	20
100	100	125	50
100	100	115	30
110	80	130	10
100	100	115	60

100	100	110	40
120	60	140	20
130	40	135	10
100	100	155	0
110	80	130	20
130	40	150	0
100	100	140	20
100	100	120	20
100	100	140	0
100	100	135	10
100	100	130	80
100	100	130	40
100	100	120	60
110	80	165	0
100	100	105	80
100	100	145	40
110	80	130	40
100	100	125	10
100	100	125	60
110	80	130	0
130	40	165	0
110	80	140	30
130	40	140	10
100	100	120	80
130	40	140	0
100	100	150	40
110	80	135	60
130	40	145	20
100	100	105	90
110	80	135	0
110	80	115	30
100	100	115	80
120	60	150	0
100	100	150	20
100	100	165	0
110	80	120	20
100	100	135	20
110	80	120	60
100	100	130	20
100	100	135	40
100	100	115	50
100	100	125	80
100	100	135	60
110	80	120	30
100	100	160	20
100	100	140	60

100	100	110	50
110	80	140	0
110	80	150	0
130	40	135	20
100	100	150	0
110	80	160	0
100	100	125	20
110	80	170	0
100	100	115	40

Table S4: Payoffs for the dictators (self) and receiver (other) for the two options in Tasks 2 & 3. Points were converted to Swiss Francs at the end of the experiment.
